# Switching from Aromatase Inhibitors to Dual Targeting Flavonoid-Based Compounds for Breast Cancer Treatment

**DOI:** 10.3390/molecules28073047

**Published:** 2023-03-29

**Authors:** Silvia Gobbi, Silvia Martini, Riccardo Rozza, Angelo Spinello, Jessica Caciolla, Angela Rampa, Federica Belluti, Nadia Zaffaroni, Alessandra Magistrato, Alessandra Bisi

**Affiliations:** 1 Department of Pharmacy and Biotechnology, Alma Mater Studiorum, University of Bologna, Via Belmeloro 6, 40126 Bologna, Italy; 2 Molecular Pharmacology Unit, Fondazione IRCSS Istituto Nazionale dei Tumori, Via Amadeo 42, 20113 Milano, Italy; 3 National Research Council of Italy Institute of Materials (CNR-IOM) c/o SISSA, Via Bonomea 265, 34136 Trieste, Italy; 4 Department of Biological, Chemical and Pharmaceutical Sciences and Technologies, University of Palermo, Viale delle Scienze, 90128 Palermo, Italy

**Keywords:** homoisoflavones, aromatase inhibitors, ERα ligands, multitarget, molecular dynamics

## Abstract

Despite the significant outcomes attained by scientific research, breast cancer (BC) still represents the second leading cause of death in women. Estrogen receptor-positive (ER+) BC accounts for the majority of diagnosed BCs, highlighting the disruption of estrogenic signalling as target for first-line treatment. This goal is presently pursued by inhibiting aromatase (AR) enzyme or by modulating Estrogen Receptor (ER) α. An appealing strategy for fighting BC and reducing side effects and resistance issues may lie in the design of multifunctional compounds able to simultaneously target AR and ER. In this paper, previously reported flavonoid-related potent AR inhibitors were suitably modified with the aim of also targeting ERα. As a result, homoisoflavone derivatives **3b** and **4a** emerged as well-balanced submicromolar dual acting compounds. An extensive computational study was then performed to gain insights into the interactions the best compounds established with the two targets. This study highlighted the feasibility of switching from single-target compounds to balanced dual-acting agents, confirming that a multi-target approach may represent a valid therapeutic option to counteract ER+ BC. The homoisoflavone core emerged as a valuable natural-inspired scaffold for the design of multifunctional compounds.

## 1. Introduction

As reported by Cancer Facts & Figures 2022 [1], breast cancer (BC) remains the most widespread tumor type and the second leading cause of cancer-related death among women after lung cancer [1]. Estrogen receptor positive (ER+) BC represents approximately 75% of all BC diagnosed. Estrogens are thus pivotal oncogenic signalling factors, playing a major role in inducing cancer cell proliferation and tumor progression. Consequently, antiestrogen therapy, focused on the silencing of estrogen signalling, represents the first-choice treatment for these BC patients after surgery. At present, the main strategies applied in antiestrogen therapy include (1) selective ER modulators (SERMs), (2) aromatase inhibitors (AIs), (3) selective ER downregulators (SERDs), and (4) gonadotropin-releasing hormone agonists (to suppress the ovarian function), or a combination of two or more of these agents [2]. SERMs (e.g., tamoxifen, raloxifene) are compounds that modulate the transcriptional activity of the receptor by competing with estrogens in ERα binding. They exert antagonistic effects in breast cancer cells while maintaining a pro-estrogenic behavior in bone and endometrial tissues. This modulatory activity is probably due to differences in the molecular and 3D structures of the co-activators and co-suppressors, which control the transcriptional activity of ERs [3]. Currently used SERMs include triphenylethylenes (tamoxifen and tamoxifen-like agents), benzothiophenes (raloxifene and arzoxifene), tetrahydronaphthalenes (lasofoxifene), and phenylindoles (basedoxifene and pipindoxifene). SERDs (e.g., fulvestrant) have antagonistic effects on ERs and lead to ERα downregulation by inducing its degradation. Indeed, the binding of a SERD to ER inhibits the recruitment of co-activators and interferes with the dimerization and nuclear localization of the receptor [4]. SERDs have greater ER-antagonistic effects with respect to SERMs, showing higher growth-inhibitory activity, but lead to substantial side effects on bone and endometrial tissues.

Aromatase inhibitors (AIs) are compounds able to bind to cytochrome P450 aromatase (AR), a key enzyme for estrogen biosynthesis. This blocks the activity of the enzyme, causing a radical decrease in estrogen levels throughout the body. To date, third-generation AIs (letrozole, anastrozole, and exemestane), together with SERMs, represent the first-line therapy for the treatment of ER+ BC. Despite their remarkable therapeutic benefits, these compounds are characterized by several side effects and the onset of resistance. Several studies have dealt with the mechanisms involved in the resistance to antiestrogen therapies, primarily focusing on the complex functions and roles of ERα and on the interconnection between the estrogen signaling network and different cellular pathways [5,6]. These mechanisms may depend on ERα mutations, a reduction in ERα expression, increased drug metabolism and/or drug efflux via the multidrug resistance P-glycoprotein, and overexpression of antiestrogen-binding site proteins able of seizing the administered therapeutic [7]. For these reasons, research is still required for the development of new potential strategies and new compounds to fight BC. Among others, approaches aimed at increasing ER inhibition/degradation are emerging and appear worthy of further development. These include PROteolysis TArgeting Chimeras (PROTACs), selective estrogen receptor covalent antagonists (SERCAs), complete ER Antagonists (CERANs), and new oral SERM/SERD hybrids. However, one of the most feasible attempts is the combination of drugs that modulate the two crucial targets involved in this pathology, AR and ERs. Several clinical studies have combined and compared AIs and SERMs to evaluate the final effect, obtaining conflicting results [8,9,10]. In 2012, Lu et al. [11] reported the first evidence of the ability of a single compound to interact with significant activities on two targets involved in the progression of ER+ BC. This discovery paved the way for the identification of new multitarget compounds capable of overcoming the limitations of current therapeutic strategies [12]; different series of compounds have been recently developed and/or evaluated for their multipotent activity [13,14,15,16,17].

**Figure 1:**
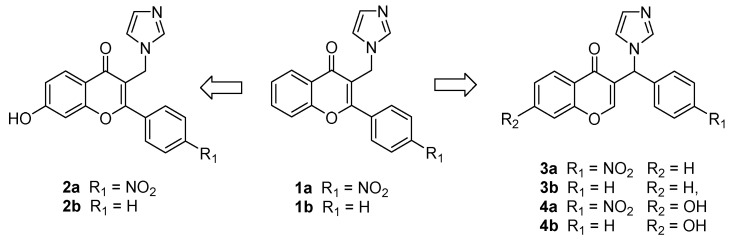
Design of the studied flavones and homoisoflavones.

Starting from the structure of our previously reported flavones **1a**,**b**, showing nanomolar AR inhibition [18], in this work we designed related molecules (Figure 1) with the aim of obtaining new dual-acting AI/SERMs compounds. In particular, considering the role of a hydroxylated function in increasing the binding affinity to ER [19] and the well-known ability of hydroxylated isoflavone phytoestrogens to modulate ERs [20], compounds **2a**,**b** were designed by inserting a hydroxyl group in position 7 of the flavone central core. This modification could also facilitate further interactions with AR enzymatic complex. In addition, we selected homoisoflavone **3a** from a series of previously obtained potent AIs [21] since it is structurally related to flavone **1a**, with the only difference being the position of the 4′-nitrophenyl ring. Exploiting this scaffold, compound **3b**, devoid of the nitro group, and the corresponding 7-hydroxylated derivatives **4a**,**b** were designed to investigate the most suitable position of the aryl moiety and the most favourable substitution pattern on the central cores of the two series for the interaction with both AR and ERα.

## 2. Results

### 2.1. Chemistry

**Scheme 1:**
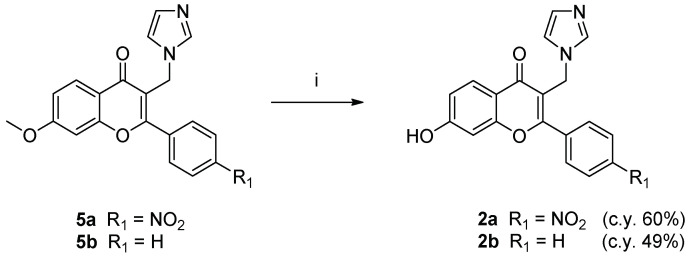
Reagent and conditions: (i) 48% HBr, reflux, 10 h.

The flavone derivatives **2a**,**b** were obtained via demethylation of the previously published **5a**,**b** [18] by treatment with 48% HBr, as shown in Scheme 1.

**Scheme 2:**
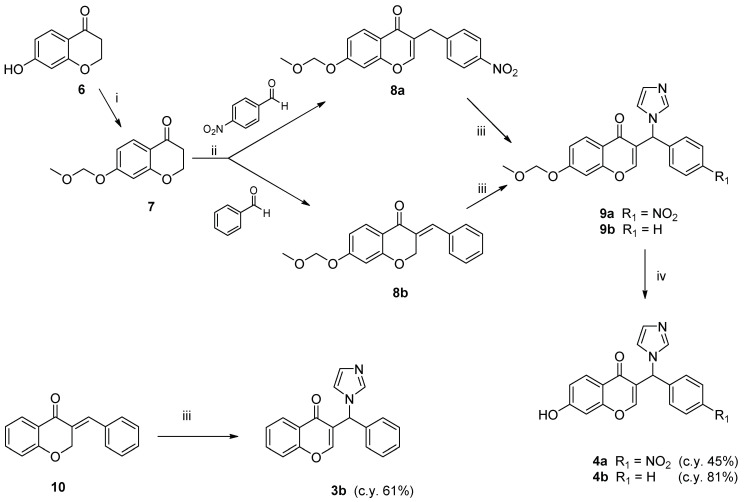
Reagent and conditions: (i) MOMCl, 60% NaH, THF, 0 °C 1 h, then rt overnight. (ii) benzaldehyde or *p*-nitrobenzaldehyde, pyridine, 150 °C, 3 h. (iii) **a**. NBS, benzoyl peroxide, CCl_4_, reflux, 5 h. **b**. imidazole, CH_3_CN, N_2_, reflux, 6 h. (iv) AcOH, H_2_SO_4_, reflux, 1 h.

**Figure 2:**
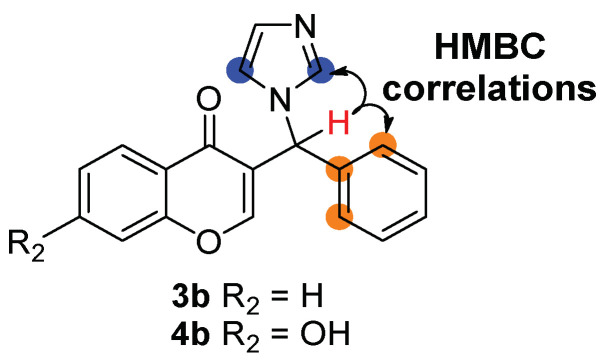
Structure determination of compounds **3b** and **4b**.

Compounds **3b** and **4a**,**b** were prepared following the synthetic strategy displayed in Scheme 2. The commercial chromanone **6** was first MOM-protected to give **7** and then condensed with the appropriate aromatic aldehyde. Generally, the main product of this condensation reaction is the *E*-3-arylidene-4-chromanone, while an exo-endo double bond migration occurs when the benzaldehyde has strong electron acceptor substituents [22]. Condensation with unsubstituted benzaldehyde led to compound **8b** while, using *p*-nitrobenzaldehyde, intermediate **8a** was the sole compound obtained. As confirmed by NMR spectra (see Supplementary Materials) and consistent with literature data [22], the positions of the endocyclic CH_2_ in **8b** and the exocyclic CH_2_ in **8a** are 5.38 ppm and 3.88 ppm, respectively. Bromination of **8a** with NBS and reaction with imidazole, followed by a deprotection step of the obtained intermediate **9a**, led to the final compound **4a**. Following the same synthetic strategy for **8b** and the commercially available compound **10**, a transposition of the double bond took place during the bromination step and the endo derivatives **4b** and **3b** were obtained, respectively, as confirmed by NMR studies. In detail, to confirm the regiochemistry of these final compounds, all protons and carbon atoms were assigned by performing ^1^H-^1^H COSY, HMBC, and HSQC spectroscopy experiments. The endo structures of **3b** and **4b** were determined by the observed HMBC correlations between the proton in red (Figure 2) and both the aromatic carbons of the phenyl group (orange) and those of the imidazole moiety (blue).

### 2.2. Biological Evaluation

The compounds were tested, at different concentrations, to assess their AR inhibitory activities by monitoring the conversion of a fluorogenic substrate of the enzyme into a highly fluorescent metabolite. AR inhibition data for letrozole were also reported as reference. Moreover, in a multitarget perspective, the binding affinity of these compounds towards ERα was also evaluated using the PolarScreen ERa Competitor Assay Kit Green (Invitrogen) by measuring their ability to prevent estrogen binding to ERα, using endoxifen as reference. The chiral homoisoflavones **3a**,**b** and **4a**,**b** were tested as racemates, aimed at evaluating whether their potential as dual inhibitors could justify subsequent enantiomer separation.

The obtained results are reported in Table 1 and show, with respect to AR inhibitory activity, that all the new compounds displayed IC_50_ values in the low- or sub-micromolar range; however, the potencies of **1a**,**b** were not improved. In particular, the introduction of a hydroxy function on the flavone core markedly decreased the activity, while it elicited a lower effect on the homoisoflavone scaffold. Considering the ERα binding affinity, homoisoflavones (**3a**,**b** and **4a**,**b**) were able to bind the receptor with IC_50_ values in the sub-micromolar range, except for **3a**. In particular, the presence of a *p*-nitro group in **3a** seemed to be detrimental with respect to the unsubstituted derivative **3b**, while the introduction of the 7-hydroxy group (**4a**) restored the affinity. On the contrary, flavones **1a**,**b** and **2a**,**b** proved not to bind to the receptor with significant affinity.

Finally, compounds **3b** and **4a** were identified as the most interesting derivatives, showing balanced and moderate potencies on both targets. Further studies are necessary to explore the ability of the compounds to modulate ERα as agonists or antagonists in view of their potential antitumor activity.

### 2.3. Molecular Dynamics Simulations

#### 2.3.1. Aromatase

In order to elucidate the structural features underlying the experimentally measured IC_50_s of the investigated compounds, we performed classical and QM/MM Molecular Dynamics (MD) simulations. These allowed us to rationalize the inhibitors’ binding mode to AR enzyme and to pinpoint their key interactions with the enzyme.

To establish the possible formation of a coordination bond between the ligands’ imidazole ring and the iron atom of the heme moiety, it is necessary to describe it at a quantum mechanical (QM) level by performing QM/MM calculations. These allow to accurately account for the metal-ligand interaction while explicitly considering its biomolecular environment [24,25]. Yet, due to the significant computational cost of this type of calculation, we selected compounds **4a** and **3b** to evaluate the impact of the nitro and hydroxyl substituents on their binding mode. The compounds possess a chiral centre, and although their activity was evaluated as racemic mixture, in the simulations both enantiomers (***R***-**4a**, ***S***-**4a**, ***R***-**3b** and ***S***-**3b**) were considered separately to predict their relative activity and provide information on which enantiomer could be the most active. Each AR/drug complex was initially relaxed by 100 ns of classical MD simulations in explicit solvent, followed by 10 ps of QM/MM MD simulations as in our previous studies [15,26,27]. A structural analysis of the computed drug/AR adducts disclosed that the coordination geometry (i.e., bond lengths and angles) of the inhibitors was not directly linked to the observed potency (Table 2), in line with previous studies [26]. Indeed, all the enantiomers fit nicely into the AR active site.

**Figure 3:**
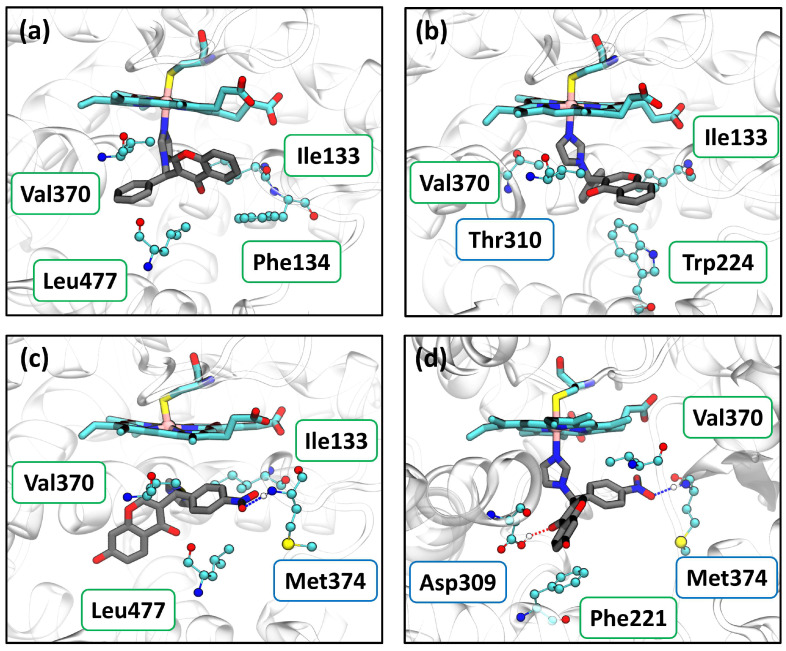
Representative structures as obtained from QM/MM molecular dynamics trajectories of the coordination complex between (**a**) ***R***-**3b**, (**b**) ***S***-**3b**, (**c**) ***R***-**4a**, and (**d**) ***S*-4a** and AR active site. AR structure is displayed in silver new cartons, the heme moiety, Cys437, and the inhibitors are shown in licorice; the residues mostly contributing to the binding are shown in balls and sticks. Labels of residues forming hydrophobic and hydrogen bonding interactions are circled in green and blue, respectively.

All compounds established stable coordination bonds with the iron atom except for ***R***-**4a,** which lost its coordination bond shortly after 2.5 ps of the QM/MM MD simulation (Figure 3). Instead, the ***S***-**4a** enantiomer**,** in addition to the coordination bond, also formed two persistent hydrogen (H)-bonds with Asp309, a key residue in the AR catalytic mechanism [29], and Met374, which is involved in the binding of the potent AR inhibitor letrozole [26] (Figure 3).

Finally, to dissect the most important intermolecular interactions established by the inhibitors with the AR catalytic site, we performed a per-residue decomposition analysis of the binding free energy (ΔG_b_) using the Molecular Mechanics Generalized Born Surface Area (MM-GBSA) method [30].

This type of calculation is performed at classical force field (FF)-level on the frames extracted from the QM/MM MD trajectories and does not account for the contribution of the inhibitor-iron coordination bond to the binding [26]. However, it can still provide insights on the important non-bonded interactions (either hydrophobic or hydrogen bonding) that each drug-candidate established with the target AR protein. This analysis revealed that all compounds preferentially engaged hydrophobic interactions with the AR binding pocket, which is indeed mostly composed of hydrophobic residues (Table 3).

Overall, **4a** displays a larger ΔG_b_ (−16.48 kcal/mol) when compared to **3b** (−10.84 kcal/mol), reflecting the experimental IC_50_ trend. This is also clearly shown by the better per-residue decomposition analysis of the ΔG_b_, where more residues contribute to the ΔG_b_ by more than −1 kcal/mol (Table 3). Since the calculated ΔG_b_s do not account for the contribution coming from the coordination bond, the ΔG_b_ value of ***R***-**4a**, the only compound not coordinating the heme moiety, is not directly comparable with those of ***R*/*S*3b** and ***S***-**4a**, which instead establish a coordination bond. This bond may indeed provide an extra stabilization, which is neglected in the calculations.

#### 2.3.2. Estrogen Receptor α

We initially performed docking simulations of all compounds to the ligand binding cavity of ERα (see Supplementary Materials, Table S1). Nevertheless, no marked differences were observed in the relative docking poses of the investigated complexes.

**Figure 4:**
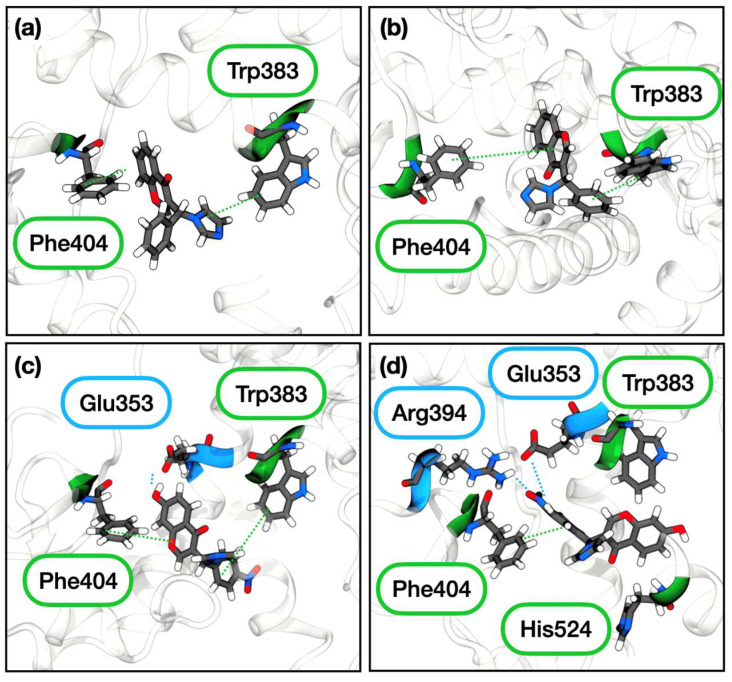
Binding poses of (**a**) ***R***-**3b,** (**b**) ***S***-**3b**, (**c**) ***R***-**4a**, and (**d**) ***S***-**4a** to the ERα ligand binding cavity as obtained from a representative cluster of 500 ns classical MD trajectory. ERα is shown as white new cartoons. The residues forming aromatic interactions are depicted in green new cartoons and those forming electrostatic interactions are displayed as blue new cartoons. The relative labels are circled in green and blue, respectively.

For the most interesting derivatives ***R*/*S***-**3b** and ***R*/*S***-**4a**, we selected the most energetically favorable docking pose for subsequent MD simulation studies. As a result, all compounds remained stably bound to the ERα ligand binding cavity lined by helices H3, H6, S2, and H1 for 500 ns of MD simulations (Figure 4) [31].

In order to identify the crucial interactions defining the binding pose of each ligand, we computed their interaction fingerprint (Table 4), discovering the type of interactions (i.e., H-bonds, charge-dipole, or aromatic interactions) [32] contributing the most to drug stability.

As a common feature, we found the presence of π-π staking interactions between the compounds’ aromatic rings and residues Trp383 and Phe404, respectively, located on helices H6 and S2. We noticed that Phe404 π-stacks with the 4*H*-chromen-4-one portion in both R enantiomers. Conversely, persistent electrostatic interactions are exclusively present in compound **4a** due to its polar substituent: the hydroxyl group H-bonds with Glu353 on helix H3 in the R enantiomer. ***S***-**4a** H-bonds with its nitro group to the same residue. Moreover, ***S***-**4a** also exhibits a charge-dipole interaction, involving its nitro group and the carboxyl group of Glu353, and π-stacks with His524 on the H11 helix.

In order to estimate the ΔG_b_ of the inhibitors to Erα, we performed MM-GBSA calculations on the 500 ns MD simulations [15,26], obtaining as average total ΔG_b_ = −28.50 ± 0.48 and −39.10 ± 0.48 kcal/mol for **3b** and **4a**, respectively. This suggests that MM-GBSA method is not sensitive enough to account for the subtle difference in the IC_50_s of these compounds on ERα. However, the per-residue decomposition sheds light on which residues contribute most to lowering the ΔG_b_ values. The results (Table 5) pinpoint the hydrophobic residues from Leu346 to Ala350, located on helix H3, residues from Leu384 to Leu391, located on helix H6, residue Phe404, located on helix S2, residue Met421, placed on H8, and residue Leu525 forming a hydrophobic cavity which mediates ligand binding as the most important ones. The only exceptions are the polar Thr347 and the negatively charged Glu353 placed on helix H3. As such, MD simulations of ERα/drug complexes revealed that compounds **3b** and **4a** are mostly stabilized by hydrophobic interactions lined by the helices H3 and H6, with an important contribution coming from residues Met421@H8 and Leu525@H11, respectively. Moreover, the π-stacking interactions with Phe404 and Trp383 and the hydrophobic interactions of Leu387 grant stability to the compounds. In particular, the ligand phenyl group position appears to be important to ameliorate ***R***-**3b** and ***S***-**4a** binding poses by increasing the stabilizing energetic contribution of Phe404.

## 3. Discussion

In this paper, a small series of derivatives was designed, aimed at obtaining dual targeting compounds to fight ER+ BC, starting from our previously reported flavone-based nanomolar AIs. To obtain binding ability to ERs, a hydroxy group was introduced, and the position of the flavone 2-phenyl ring was also modified, leading to both flavone- and homoisoflavone- derivatives. The compounds were then tested to evaluate their dual activity profiles on AR and ERα. The results proved that the new derivatives maintained activity on AR, albeit lower with respect to the previously reported compounds. A different profile was observed for the two series when binding to Erα, where none of the flavones showed appreciable affinity for the target, while the repositioning of the 2-phenyl in homoisoflavone compounds led to submicromolar activity.

In the flavone series, the introduction of the 7-hydroxy group appeared detrimental for AR inhibition, leading to a 50-fold reduction in IC_50_ values, with the same trend for nitro- and hydrogen-bearing compounds (**2a** and **2b** vs. **1a** and **1b**, respectively). The higher activities observed for the nitro-substituted **1a** and **2a**, with respect to the corresponding unsubstituted derivatives, allowed us to assess the positive contribution of the nitro group for an appropriate interaction with AR. Unfortunately, flavones **1a** and **1b** proved not to bind efficiently to ERα, and the introduction of the hydroxy group on their scaffold did not seem to have a positive effect on receptor targeting, with **2a** and **2b** also inactive up to 10 µM.

A different behavior can be seen for the homoisoflavone derivatives. AR inhibition was 10-fold increased by the presence of a nitro substituent on the phenyl ring (**3a** and **4a**), while the introduction of the hydroxy function on the scaffold (**4a** and **4b**) again proved to negatively affect potency, as observed in the flavone series but to a lesser extent. On the contrary, binding to ERα seemed to take advantage of the presence of the hydroxy group (**4a** and **4b**), while the nitro group proved to unfavorably affect activity. In detail, the nitro-substituted **3a** proved unable to bind to ERα up to 10 µM, while the corresponding unsubstituted **3b** showed submicromolar activity. A slight improvement could also be seen in the IC_50_ of the hydroxylated compounds **4a** and **4b**. As expected, the comparison between the inactive **3a** and **4a** highlights the prominent role of the hydroxy group in receptor binding, which can overcome the negative effect exerted by the nitro function. This trend could also explain the smaller difference in binding potency between **3b** and **4b**, both devoid of the nitro substituent.

The extensive computational studies performed on these molecules clearly showed their ability to establish appropriate interactions with the two targets. Both enantiomers of **4a** established mostly hydrophobic interactions with the AR enzyme, while for ***S***-**4a,** a coordination bond and two hydrogen bonds to Asp309 and Met374 were also observed, likely suggesting it to be the most active enantiomer. Instead, although both enantiomers of **3b** form coordination bonds with the heme iron atom, they establish weaker interactions. Conversely, when **3b** and **4a** targeted ERα, the MD simulations did not allow to discriminate the most active ligand, also owing to the more similar IC_50_. However, both **3b** enantiomers had a similar binding energy, while for **4a**, the S enantiomer seemed to be the most active.

Taken together, these results showed a different behavior for the two selected scaffolds. While the introduction of the hydroxy function surprisingly did not grant ERα binding affinity to flavone-based compounds and led to a significant reduction of AR inhibition, the shift to homoisoflavone allowed the targeting of AR and gained affinity for the receptor. Nevertheless, none of the obtained dual acting derivatives could maintain the AR inhibitory activity in the nanomolar range, nor achieve high potency on ERα. Thus, although **3b** and **4a** showed balanced activity on both targets, their potencies do not support further investigations on these molecules, even if the scaffold may represent a valuable starting point for additional modifications. Potential improvement in potency could be pursued with the introduction of substituents with different properties with respect to the electron-withdrawing nitro group on the phenyl ring, or the addition of novel functionalities able to establish strong interactions with the targets. The substitution pattern could also be revised to obtain more appropriate interactions.

## 4. Materials and Methods

### 4.1. Chemistry

#### 4.1.1. General Materials and Methods

Starting materials were used as commercial products with high-grade purity and solvents were of analytical grade. Thin layer chromatography (TLC) on precoated silica gel plates (Merck Silica Gel 60 F254, Darmstadt, Germany), visualized with a UV254 lamplight, was used to follow reaction progress. Flash chromatography was performed on a silica gel column (Kieselgel 40, 0.040–0.063 mm, Merck, Darmstadt, Germany). Melting points were determined using a Büchi apparatus and are uncorrected. ^1^H NMR and ^13^C NMR spectra were obtained with a Varian Gemini spectrometer (Scientific instruments, Palo Alto, CA, USA) (400 MHz and 101 MHz, respectively) in CDCl_3_ unless otherwise indicated. Chemical shifts (δ) are indicated as parts per million (ppm) values relative to the standard tetramethylsilane (TMS) and coupling constants (*J*) are reported in Hertz (Hz). Abbreviations for spin multiplicities are as follows: s (singlet), d (doublet), t (triplet), br (broad), q (quartet), and m (multiplet). UHPLC−MS analyses were performed using a Waters ACQUITY ARC UHPLC/MS system, a QDA mass spectrometer equipped with an electrospray ionization interface, and a 2489 UV/Vis detector operating at 254 nm and 365 nm (Waters Alliance, San Diego, CA, USA). The analyses were performed on an XBridge BEH C18 column (10 × 2.1 mm i.d., particle size 2.5 μm) with a Xbridge BEH C18 VanGuard Cartridge precolumn (5 mm × 2.1 mm i.d., particle size 1.8 μm), using H_2_O (0.1% formic acid) (A) and MeCN (0.1% formic acid) (B) as mobile phases. Linear gradient: 0−0.78 min, 20% B; 0.78–2.87 min, 20−95% B; 2.87–3.54 min, 95% B; 3.54–3.65 min, 95–20% B; 3.65–5.73, 20% B; flow rate: 0.8 mL/min. Electrospray ionization (positive and negative modes) was applied in the mass scan range 50−1200 Da. All studied compounds showed >95% purity. Compounds were named applying the naming algorithm developed by CambridgeSoft Corporation and used in ChemDraw Professional 22.0 (PerkinElmer Inc., Waltham, MA, USA).

#### 4.1.2. General Procedure I. (Synthesis of Compounds **2a**,**b**)

A solution of 7-methoxyflavone (**5a**,**b**) [18] in 48% HBr was refluxed for 10h. The reaction mixture was diluted with H_2_O, basified with 2N NaOH, and washed with DCM. The aqueous phase was then acidified with 6N HCl until a precipitate was formed that was collected by filtration, dried, and purified by flash chromatography, if necessary, to provide the final compounds **2a**,**b**.

*3-((1H-imidazol-1-yl)methyl)-7-hydroxy-2-(4-nitrophenyl)-4H-chromen-4-one* (**2a**). Using the general procedure I and starting from **5a** (0.52 g, 1.4 mmol) and 10 mL of 48% HBr, a crude compound was obtained that was purified by flash column chromatography (DCM:methanol 4.75:0.25) to give 0.30 g (60%) of **2a**, mp 260 °C. ^1^H NMR (400 MHz, DMSO-*d*_6_) δ 10.97 (br, 1H), 8.44–8.35 (m, 2H), 8.01–7.89 (m, 3H), 7.49 (s, 1H), 7.04–6.93 (m, 2H), 6.88 (d, *J* = 2.2 Hz, 1H), 6.81 (s, 1H), 4.95 (d, *J* = 14.8 Hz, 2H). ^13^C NMR (101 MHz, DMSO-*d*_6_) δ 175.4, 163.3, 162.0, 157.6, 148.7, 137.6, 137.1, 130.4 (2C), 128.0, 127.0, 124.0 (2C), 119.2, 116.6, 115.7, 115.2, 102.4, 41.0. MS (ES) *m*/*z*: 364 (M + 1).

*3-((1H-imidazol-1-yl)methyl)-7-hydroxy-2-phenyl-4H-chromen-4-one* (**2b**). Using the general procedure I and starting from **5b** (0.69 g, 2.1 mmol) and 13 mL of 48% HBr, 0.33 g (49%) of **2b** were obtained, mp 276–278 °C. ^1^H NMR (400 MHz, DMSO-*d*_6_) δ 10.91 (br, 1H), 7.95 (d, *J* = 8.8 Hz, 1H), 7.67–7.55 (m, 5H), 7.43 (s, 1H), 6.97 (dd, *J* = 8.8, 2.3 Hz, 1H), 6.92 (br, 1H), 6.88 (d, *J* = 2.2 Hz, 1H), 6.80 (br, 1H), 4.93 (s, 2H). ^13^C NMR (101 MHz, DMSO-*d*_6_) δ 175.7, 164.3, 163.1, 157.6, 144.2, 131.8, 131.6, 131.0, 128.9, 128.6, 127.0, 121.4, 115.7, 115.5, 115.2, 102.4, 40.1. MS (ES) *m*/*z*: 319 (M + 1).

#### 4.1.3. Synthesis of 7-(methoxymethoxy)chroman-4-one (**7**)

To a suspension of NaH (60% dispersion in mineral oil, 0.88 g, 22.1 mmol) in dry THF (16 mL) at 0 °C under N_2_ atmosphere, a solution of **6** (3.0 g, 18.4 mmol) in THF was added. Then, MOM-Cl (1.78 g, 22.1 mmol) was added, and the resulting mixture was stirred at 0 °C for 1 h and at room temperature overnight. The reaction mixture was poured into ice and extracted with DCM; the organic layer was washed with 2N NaOH, dried over Na_2_SO_4_ and evaporated to give 1.88 g (49%) of **7** as an oil. ^1^HNMR δ 7.88 (d, *J* = 8.8 Hz, 1H), 6.70 (dd, *J* = 2.0 and 8.8 Hz, 1H), 6.61 (d, *J* = 2.0 Hz, 1H), 5.22 (s, 2H), 4.52 (t, *J* = 6.4 Hz, 2H), 3.51 (s, 3H), 2.75 (t, *J* = 6.4 Hz, 2H).

#### 4.1.4. General Procedure II. (Synthesis of Compounds **8a**,**b**)

A mixture of **7** (1.0 eq) and benzaldehyde or 4-nitrobenzaldehyde (1.0 eq) in piperidine was heated at 150 °C for 3 h. The reaction mixture was diluted with DCM and washed with H_2_O. The organic phase was dried over Na_2_SO_4_ and evaporated. The crude compounds were purified by crystallization from ligroin.

*7-(methoxymethoxy)-3-(4-nitrobenzyl)-4H-chromen-4-one* (**8a**). Using the general procedure II and starting from **7** (1.53 g, 7.4 mmol) and 4-nitrobenzaldehyde (1.12 g, 7.4 mmol) in 0.11 mL of piperidine, 1.16 g of **8a** (46%) were obtained, mp 137–141 °C. ^1^HNMR δ 8.18 (m, 3H), 7.71 (s, 1H), 7.45 (d, *J* = 8.4 Hz, 2H), 7.08–7.01 (m, 2H), 5.28 (s, 2H), 3.88 (s, 2H), 3.52 (s, 3H).

*(E)-3-benzylidene-7-(methoxymethoxy)chroman-4-one* (**8b**). Using the general procedure II and starting from **7** (1.75 g, 8.4 mmol) and benzaldehyde (0.89 g, 8.4 mmol) in 0.18 mL of piperidine, 0.85 g of **8b** (40%) were obtained, mp 70–72 °C. ^1^HNMR δ 7.98 (d, *J* = 8.8 Hz, 1H), 7.86 (s, 1H), 7.55–7.40 (m, 3H), 7.35–7.23 (m, 2H), 6.74 (d, *J* = 2.0 and 8.8 Hz, 1H), 6.61 (d, *J* = 2.0 Hz, 1H), 5.38 (s, 2H), 5.21 (s, 2H), 3.51 (s, 3H).

#### 4.1.5. General Procedure III (Synthesis of Compounds **9a**,**b** and **3b**)

To a solution of **8a, 8b**, or **10** (1.0 eq) in CCl_4_, N-bromosuccinimide (1.0 eq) and a catalytic amount of benzoyl peroxide were added. The mixture was refluxed for 5 h, then hot filtered and evaporated to dryness to give the brominated intermediates that were reacted, without further purification, with imidazole (3.0 eq) in dry acetonitrile, under N_2_ atmosphere for 6 h. The reaction mixture was evaporated to dryness and purified by flash column chromatography, using a suitable eluent, to give intermediates **9a** and **9b** and the final compound **3b**.

*3-((1H-imidazol-1-yl)(4-nitrophenyl)methyl)-7-(methoxymethoxy)-4H-chromen-4-one* (**9a**). Using the general procedure III and starting from **8a** (1.16 g, 3.4 mmol), a crude compound was obtained that was purified by flash column chromatography (toluene: acetone 3:2) to give 0.62 g (45%) of **9a** as an oil. ^1^HNMR δ 8.25 (d, *J* = 8.4 Hz, 2H), 8.15(d, *J* = 8.8 Hz, 1H), 7.58 (s, 1H), 7.45 (s, 1H), 7.38 (d, *J* = 8.4 Hz, 2H), 7.28–7.11 (m, 3H), 6.98 (s, 1H), 6.85 (s, 1H), 5.28 (s, 2H), 3.50 (s, 3H).

*3-((1H-imidazol-1-yl)(phenyl)methyl)-7-(methoxymethoxy)-4H-chromen-4-one* (**9b**). Using the general procedure III and starting from **8b** (0.85 g, 2.8 mmol), a crude compound was obtained that was purified by flash column chromatography (petroleum ether: ethyl acetate 1:4) to give 0.39 g (39%) of **9b** as an oil. ^1^HNMR δ 8.17 (d, *J* = 8.8 Hz, 1H), 7.55 (s, 1H), 7.41–7.35 (m, 4H), 7.19–7.02 (m, 5H), 6.92 (s, 1H), 6.81 (s, 1H), 5.28 (s, 2H), 3.50 (s, 3H).

*3-((1H-imidazol-1-yl)(phenyl)methyl)-4H-chromen-4-one* (**3b**). Using the general procedure III and starting from **10** (0.90 g, 3.8 mmol), a crude compound was obtained that was purified by flash column chromatography (ethyl acetate) to give 0.70 g (61%) of **3b**, mp 120–122 °C. ^1^H NMR (400 MHz, DMSO-*d*_6_) δ 8.05 (d, *J* = 7.6 Hz, 1H), 7.91 (s, 1H), 7.84 (t, *J* = 7.3 Hz, 1H), 7.79 (s, 1H), 7.68 (d, *J* = 8.4 Hz, 1H), 7.52 (t, *J* = 7.5 Hz, 1H), 7.40–7.32 (m, 3H), 7.28 (s, 1H), 7.18 (d, *J* = 7.1 Hz, 2H), 6.96 (s, 1H), 6.84 (s, 1H). ^13^C NMR (101 MHz, DMSO-*d*_6_) δ 175.0, 156.5, 155.8, 138.2, 137.5, 134.6, 128.7 (2C), 128.5, 128.0, 127.1 (2C), 125.8, 125.1, 123.2, 123.0, 119.5, 118.5, 56.0. MS (ES) *m*/*z*: 303 (M + 1).

#### 4.1.6. General Procedure IV. (Synthesis of Compounds **4a**,**b**)

A solution of chromen-4-one (**9a,b**,1.0 eq) in acetic acid/H_2_O (1:1) and H_2_SO_4_ (0.04 mL) was refluxed for 1.5 h. The reaction was then poured into ice and neutralized with K_2_CO_3_. A solid was formed that was collected by filtration and dried to give compounds **4a**,**b**.

*3-((1H-imidazol-1-yl)(4-nitrophenyl)methyl)-7-hydroxy-4H-chromen-4-one* (**4a**). Using the general procedure IV and starting from **9a** (0.39 g, 0.96 mmol) in 10 mL of diluted acetic acid, 0.16 g (45%) of **4a** were obtained, mp 190–193 °C (dec). ^1^HNMR (400 MHz, DMSO-*d*_6_) δ 8.21 (d, *J* = 9.2 Hz, 2H), 7.96 (s, 1H), 7.86 (d, *J* = 8.8 Hz, 1H), 7.82 (s, 1H), 7.36 (d, *J* = 8.4 Hz, 2H), 7.30 (s, 1H), 6.98 (s, 1H), 6.94 (s, 1H), 6.92 (d, *J* = 5.2 Hz, 1H), 6.86 (s, 1H). ^13^C NMR (101 MHz, DMSO-*d*_6_) δ 175.0, 165.0, 157.2, 155.9, 147.1, 146.1, 137.7, 128.9, 128.2 (2C), 125.9, 125.3, 123.8 (2C), 123.3, 119.4, 118.6, 101.6, 55.9. MS (ES) *m*/*z*: 364 (M + 1).

*3-((1H-imidazol-1-yl)(phenyl)methyl)-7-hydroxy-4H-chromen-4-one* (**4b**). Using the general procedure IV and starting from **9b** (0.34 g, 1.0 mmol) in 11 mL of diluted acetic acid, 0.26 g (81%) of **4b** were obtained, mp 167–169 °C (dec). ^1^HNMR (400 MHz, DMSO-*d*_6_) δ 7.74 (s, 1H), 7.68 (d, *J* = 8.9 Hz, 1H), 7.61 (s, 1H), 7.39–7.29 (m, 3H), 7.24 (s, 1H), 7.13 (d, *J* = 7.2 Hz, 2H), 6.93 (s, 1H), 6.76 (s, 1H), 6.70 (d, *J* = 8.7 Hz, 1H), 6.55 (s, 1H). ^13^C NMR (101 MHz, DMSO-*d*_6_) δ 173.6, 170.0, 158.6, 154.7, 138.8, 137.5, 128.6 (2C), 128.4, 127.7, 126.9 (2C), 126.0, 122.0, 119.5, 118.2, 112.3, 101.9, 56.1. MS (ES) *m*/*z*: 319 (M + 1).

### 4.2. Biological Evaluation

#### 4.2.1. Aromatase Inhibition Assay

The in vitro aromatase inhibitory activity of the novel compounds was evaluated using Aromatase Inhibitor (CYP19A) Screening Kit (Fluorometric, Bio Vision) according to the manufacturer’s instructions. This assay employs a fluorogenic aromatase substrate that is converted into a highly fluorescent metabolite detected in the visible range (Ex/Em = 488/527 nm). Serial dilutions of test compounds (0.0001–10 µM) were dissolved in DMSO and the results were compared to letrozole (1 μM), used as a reference compound. Samples and recombinant human aromatase were added to the plate and incubated for 10 min at 37 °C. The activity was measured immediately after the addition of the aromatase substrate/NADP+ mixture.

#### 4.2.2. Estrogen Receptor α Binding Assay

PolarScreen ERα Competitive Assay Kit, Green (Cat.No15883, Invitrogen, Waltham, MA, USA) was used to determine the relative affinity of novel compounds for ERα. The protocol provided by the manufacturer was followed. Briefly, purified ERα (75 nM) was incubated with serial dilutions of test compounds (0.00001 µM to 1 µM) and Fluormone^TM^ ES2 (4.5 nM). After 2 h incubation at room temperature, fluorescence polarization was measured using a Tecan Spark Plate Reader with 485 nm excitation and 530 nm emission interference filters. All measurements were conducted in triplicate and IC_50_ values were calculated using GraphPad Prism software (GraphPad Software version 6.0c).

### 4.3. Computational Details

#### 4.3.1. Docking Calculations

Docking simulations of compounds ***R***-**3b**, ***S***-**3b**, ***R***-**4a**, and ***S***-**4a** on aromatase (AR) and all compounds on the Estrogen Receptor (ER)α monomer were performed with Glide 8.9 [33] using the extra precision (EP) protocol. AR and ERα monomer structures were extracted from a representative cluster obtained from MD simulations validated in our previous studies [29,31]. For ERα, we started from a structure of the ligand binding cavity in the antagonist conformation, as in previous studies [31].

To mimic protein flexibility, we used a van der Waals (vdW) radius scaling factor of 0.80 Å for protein and ligands atoms having a partial charge less than 0.15. Finally, in the case of AR, a metal constraint was considered in order to obtain binding poses where the nitrogen atom was at coordination distance from the heme iron, as performed in previous studies [15,26]. Moreover, the possibility of tautomerism of the imidazole ring of all the investigated compounds has been explicitly considered.

#### 4.3.2. Model Building

We used the PropKa 3.1 software to determine the protonation state of ionizable residues [34]. In the case of AR models, Asp309 was considered its neutral form, as previously reported in literature [35]. All system topologies were built using the tleap module of AmberTools20 [36]. The FF14SB AMBER force field (FF) [37] was used for the protein description, the Shahrokh et al. parameters for the heme moiety and Cys437 [38], and the general Amber FF (GAFF) for the inhibitors [39]. In all the systems, we used a physiological concentration of 0.15 M in NaCl salt concentration. The required number of ions was obtained exploiting the SLTCAP webserver [40]. Na^+^ and Cl^−^ ions were described using the Joung and Cheatham parameters [41]. For all the drugs investigated, we computed the ESP charges performing geometry optimization at Hartree–Fock level of theory with a 6–31G* basis set using the Gaussian 09 software. Subsequently, we generated RESP charges through the Antechamber module of Ambertools22 [42]. A layer of 14 Å from the solute boundary of TIP3P water molecules has been added to all systems [43], leading to a total system size of ~66,342 and ~76,000 atoms for AR and ER, respectively. Finally, the topologies were converted to the GROMACS 2020.6 format using the parmed software [44].

#### 4.3.3. Classical MD Simulations

We ran classical MD simulations using GROMACS 2020.6 software [45], using a 2fs integration time step. To constrain covalent bonds involving hydrogen atoms, we utilized the LINCS algorithm [46], and for electrostatic interactions we employed the Particle Mesh Ewald scheme with a 10 Å real space cut-off [47]. The MD simulations were performed in the isothermal-isobaric NPT ensemble at 300 K, with the velocity-rescaling thermostat [48] and the Parrinello–Rahman barostat [49]. Prior to the molecular dynamics simulations, we carried out a system energy minimization using the steepest descent algorithm, and then gradually heated the system to 300 K over 12 ns, with 50 K increments every 2 ns. During this process, we kept the entire system highly restrained, except for the solute hydrogens and solvent atoms. After this, we switched to the NPT ensemble, scaling the pressure to 1 bar, and used two different barostats: the Berendsen barostat for 20 ns, with the same restraints on the solute atoms, and the Parrinello–Rahman barostat for an additional 30 ns, leaving the residues side chains free to move. We gradually decreased the restraints in 20 ns. In the end, we ran MD simulations of 100 and 500 ns for AR and ERα models, respectively, in order to relax the structure of AR and ERα in the presence of inhibitors, and to maintain the stability of the coordination bond between the heme iron and the imidazole ring of the selected molecules.

#### 4.3.4. QM/MM Molecular Dynamics Simulations

Due to the difficulties in accurately describing coordination bonds with classical force fields [50], we performed QM/MM Born Oppenheimer MD simulations using the CP2K 7.1 program [51] for AR-inhibitors complexes. The QM region of each system consisted of the heme group, Cys437, and the imidazole ring of the inhibitors (54 atoms) and was simulated using a 20 Å cubic box. We used Density Functional Theory (DFT) with the BLYP exchanges correlation functional and a dual Gaussian-type/Plane Waves basis set (GPW) [52]. Specifically, we employed a double ζ (MOLOPT) basis set, an auxiliary PW basis set with a density cutoff of 400 Ry, and Goedecker–Teter–Hutter (GTH) pseudopotentials [53]. This QM/MM MD simulation protocol has been successfully adopted in previous biomolecular simulation studies also involving AR [54,55]. We used an integration step of 0.5 fs in all the QM/MM MD simulations under the NVT ensemble, with the dangling bonds between the quantum and classical layers saturated using capping hydrogen atoms. All the systems were optimized and equilibrated at 300 K without constraints for 2 ps, using a Nosé-Hoover thermostat [56]. After equilibration, 8 ps of QM/MM MD simulations were performed, for a total simulation time of 10 ps. Similar to previous studies [57], we used a doublet spin state for the heme iron, and the MM region was described using the same force field as the classical MD simulations.

#### 4.3.5. Analysis

The cpptraj module of AmberTools22 [42] was used to compute the root-mean-square fluctuation (RMSF), root-mean-square deviation (RMSD), radius of gyration (Rg), and hydrogen (H)-bond analysis. The interaction framework was analyzed through prolif packages [58], while cluster analysis of the MD trajectories was performed via the software GROMACS 2020.6 [59] using the Daura et al. algorithm [45].

The Molecular Mechanics Generalized Born Surface Area (MM-GBSA) free energy calculations were carried out using the MM_PBSA.py tool of Amber20 [60] on 100 equispaced frames taken from the last 5 ps of the QM/MM MD trajectories (for AR) and from 500 ns of the ERα/drugs equilibrated trajectories. In these calculations, a generalized born solvation model was employed (igb = 8) with a salt concentration of 0.150 M, using a value of 4 for the internal dielectric constant of the protein and keeping the default value of 80 for the external dielectric constant. The free energy conformational entropic component was not considered, as it has been previously suggested that this term does not improve the quality of the results when using the MM-G(P)BSA method [61,62]. The MD trajectories visualization and their images creation were performed using the VMD software [63].

## 5. Conclusions

In this study, we took advantage of our previously reported flavone-based nanomolar AR inhibitors for the design of new dual-acting AI/SERMs compounds. The structure was appropriately modified to achieve ERα binding affinity, thus switching from single- to multitarget compounds. While the potency on AR was not increased, some compounds were identified that showed submicromolar affinity for ERα. In a multitarget perspective, the obtained results identified homoisoflavones **3b** and **4a** as well-balanced submicromolar dual-acting compounds. Despite the non-optimal potency of these molecules, the encouraging results point at this scaffold as an unexplored source of novel agents targeting both AR and ERα. This study highlighted the feasibility of exploiting natural-inspired molecules to obtain multipotent compounds, simultaneously engaging different targets involved in ER+ BC. These results build on and expand recent efforts for the design of dual targeting AR and ERα agents and supply further evidence that this approach could represent a viable strategy in the fight against ER+ BC.

## Figures and Tables

**Table 1:** Biological profiles of the studied compounds: inhibition of AR activity and ERα binding.

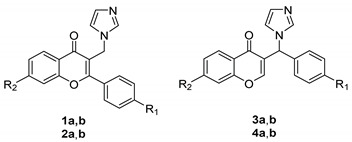				
Compound	R_2_	R_1_	AR Inhibition IC_50_ μM ^1^	ERα Binding IC_50_ μM ^1^
**1a**	H	NO_2_	0.045 ^2^	>10
**1b**	H	H	0.072 ^2^	>10
**2a**	OH	NO_2_	2.1	>10
**2b**	OH	H	4.0	>10
**3a** ^3^	H	NO_2_	0.063	>10
**3b**	H	H	0.50	0.310
**4a**	OH	NO_2_	0.122	0.595
**4b**	OH	H	2.2	0.203
**Letrozole**	-	-	0.005	>10
**Endoxifen**	-	-	>10	0.043

^1^ Data represent the mean values of at least three independent experiments performed in triplicate. The deviations were within ±5%. ^2^ Ref. [18], ^3^ Ref. [23].

**Table 2:** Structural parameters for the binding of the inhibitors **4a** and **3b** to the AR active site. The coordination bonds (Å) and angles (deg) of the imidazole moieties of the ligands to the heme iron atom are reported. The clinically used inhibitor letrozole (LTZ) is also shown for comparison. Data for LTZ refer to reference [28].

	IC_50_ (µm)	Distance (Fe-N) Å	Angle (Planes) Deg
**LTZ**	0.010	2.33 ± 0.15	91.8 ± 2.7
***R***-**3b**	0.50 ^1^	2.23 ± 0.10	94.7 ± 2.2
***S***-**3b**	0.50 ^1^	2.16 ± 0.08	87.0 ± 2.2
***R***-**4a**	0.122 ^1^	-	-
***S***-**4a**	0.122 ^1^	2.30 ± 0.12	93.2 ± 2.7

^1^ Data refer to the racemate.

**Table 3:** IC_50_ (µM) for the measured inhibition of the Aromatase enzymatic activity. Binding free energy (ΔG_b_, kcal/mol), along with its per-residue decomposition, obtained using the Molecular Mechanics Generalized Surface Area (MM-GBSA) method for the binding of compounds ***R*/*S***-**3b** and ***R*/*S***-**4a** to AR as obtained from QM/MM molecular dynamics trajectories. The corresponding standard error of mean to the ΔG_b_ is reported. Residues involved in stabilizing the inhibitor binding pose are marked in yellow, in light green, and in dark green when their contribution to ΔG_b_ is smaller than −1.0 kcal mol, when it ranges between −1.01 and −1.50 kcal/mol, and when it larger than −1.51 kcal/mol, respectively.

	*R*-3b	*S*-3b	*R*-4a	*S*-4a
**IC_50_s**	0.50		0.12	
**MM-GBSA ΔG_b_**	−10.95 ± 0.93	−10.74 ± 1.05	−18.24 ± 2.06	−14.73 ± 0.96
**Average**	−10.84		−16.48	
**ΔG_b_ per-residue**				
**Arg115**	−0.70 ± 0.08	−0.54 ± 0.03	−0.95 ± 0.04	−1.15 ± 0.03
**Ile133**	−1.77 ± 0.03	−1.53 ± 0.04	−1.13 ± 0.03	−1.27 ± 0.02
**Phe134**	−1.10 ± 0.04	−0.99 ± 0.03	−0.65 ± 0.02	−0.76 ± 0.02
**Phe221**	−0.57 ± 0.03	−0.32 ± 0.02	−1.07 ± 0.03	−1.52 ± 0.04
**Trp224**	−0.89 ± 0.03	−1.52 ± 0.03	−0.68 ± 0.02	−1.01 ± 0.03
**Asp309**	−0.16 ± 0.03	−0.26 ± 0.03	−0.60 ± 0.04	−1.33 ± 0.07
**Thr310**	−0.46 ± 0.05	−1.42 ± 0.03	−1.14 ± 0.05	−1.03 ± 0.06
**Val370**	−1.75 ± 0.04	−1.49 ± 0.05	−1.90 ± 0.04	−1.28 ± 0.03
**Val373**	−0.07. ± 0.03	−0.14 ± 0.01	−1.13 ± 0.02	−1.30 ± 0.02
**Met374**	−0.90 ± 0.05	−0.59 ± 0.02	−1.18 ± 0.02	−1.15 ± 0.02
Leu477	−1.64 ± 0.03	−0.81 ± 0.04	−1.35 ± 0.03	−1.23 ± 0.03

**Table 4:** Fingerprint of the interactions that ***R***-**3b**, ***S***-**3b**, ***R***-**4a**, and ***S***-**4a** establish to ERα during 500 ns MD simulations. The residues establishing hydrophobic (π-stacking) and electrostatic interactions are reported in green and in cyan, respectively. The persistence (%) of the interaction occurrence during the MD simulations is reported.

	*R*-3b	*S*-3b	*R*-4a	*S*-4a
**Glu353**			92	90
**Trp383**	29	51	31	15
**Arg394**				98
**Phe404**	41	18	39	62
**His524**				17

**Table 5:** IC_50_ (µM) of the compounds measured on ERα. Binding free energy (ΔG_b_, kcal/mol), along with its per-residue decomposition, obtained with Molecular Mechanics Generalized Surface Area (MM-GBSA) method, along with their standard error of mean, for the binding of both enantiomers of compounds **3b** and **4a** to ERα monomer as obtained from 100 frames of the 2 μs molecular dynamics trajectories. Residues involved in stabilizing the inhibitor binding pose are marked in yellow, in light green, and in dark green when their contribution to ΔG_b_ is smaller than −1.0 kcal/mol, when it ranges between −1.01 and −1.50 kcal/mol, and when it is larger than −1.51 kcal/mol, respectively. Docking score values are reported in kcal/mol.

	*R*-3b	*S*-3b	*R*-4a	*S*-4a
**IC_50_**	0.310		0.595	
**Docking score**	−9.84	−8.68	−10.49	−8.96
**MM-GBSA ΔG_b_**	−29.36 ± 0.26	−27.64 ± 0.38	−30.17 ± 0.37	−48.03 ± 0.30
**Average**	−28.50 ± 0.46		−39.10 ± 0.48	
**ΔG_b_, Per-Residue Decomposition**				
**Met343**				−0.66 ± 0.03
**Leu346**	−0.72 ± 0.05	−1.50 ± 0.12	−0.88 ± 0.05	−2.00 ± 0.05
**Thr347**	−1.74 ± 0.06	−0.63 ± 0.05	−0.71 ± 0.04	−1.27 ± 0.03
**Leu349**	−0.51 ± 0.03	−0.56 ± 0.03	−1.07 ± 0.03	−0.97 ± 0.02
**Ala350**	−0.88 ± 0.03	−1.31 ± 0.05	−1.81 ± 0.05	−1.43 ± 0.03
**Glu353**	−2.04 ± 0.05	−0.74 ± 0.12	−1.58 ± 0.09	
**Trp383**		−0.90 ± 0.05	−0.90 ± 0.04	
**Leu384**	−1.06 ± 0.05	−1.02 ± 0.05	−1.69 ± 0.04	−0.63 ± 0.03
**Leu387**	−2.50 ± 0.06	−1.60 ± 0.06	−0.96 ± 0.05	−1.71 ± 0.04
**Met388**	−1.33 ± 0.06	−0.72 ± 0.05	−0.65 ± 0.04	−0.59 ± 0.03
**Leu391**	−1.00 ± 0.04	−0.57 ± 0.07	−0.89 ± 0.04	−0.93 ± 0.02
**Phe404**	−1.96 ± 0.07	−0.69 ± 0.07	−1.15 ± 0.03	−1.54 ± 0.03
**Val418**	−0.51 ± 0.03	−1.11 ± 0.09		
**Glu419**		−0.96 ± 0.20		
**Gly420**		−0.61 ± 0.06		
**Met421**	−1.61 ± 0.06	−1.69 ± 0.07	−1.76 ± 0.06	−0.90 ± 0.03
**Met522**			−0.51 ± 0.04	
**Leu525**	−1.11 ± 0.05	−0.83 ± 0.04	−2.12 ± 0.05	−1.94 ± 0.05
**Met528**				−1.05 ± 0.08

## Data Availability

Docking poses and representative structures of compounds ***R*/*S***-**3b** and ***R*/*S***-**4a** bound to AR and ERα re available upon request to the authors.

## Supplementary Materials

The following supporting information can be downloaded at: https://www.mdpi.com/article/10.3390/molecules28073047/s1, Figures S1–S9: NMR spectra of compounds **2a**,**b**, **3b**, **4a**,**b**; Figure S10: NMR of intermediates **8a** and **8b**; Table S1: Docking score of all compounds to ERα.

## Sample Availability

Samples of the compounds are available from the authors.
