# Supplementary material for: Switching from Aromatase Inhibitors to Dual Targeting Flavonoid-Based Compounds for Breast Cancer Treatment
Source: Molecules. 2023 Mar 29;28(7):3047. doi: 10.3390/molecules28073047 (PMC10096035; doi:10.3390/molecules28073047)
Supplement: Supplementary file 1 [file molecules-28-03047-s001.zip › molecules-2272138-supplementary.pdf]

Supplementary Materials

Switching from Aromatase Inhibitors to Dual Targeting Flavonoid-Based Compounds for Breast Cancer Treatment

Silvia Gobbi [1,*], Silvia Martini [2], Riccardo Rozza [3], Angelo Spinello [4], Jessica Caciolla [1], Angela Rampa [1], Federica Belluti [1], Nadia Zaffaroni [2], Alessandra Magistrato [3] and Alessandra Bisi [1,*]

[1]*Department of Pharmacy and Biotechnology, Alma Mater Studiorum-University of Bologna, Via Belmeloro 6, 40126 Bologna, Italy*

[2]*Molecular Pharmacology Unit, Fondazione IRCSS Istituto Nazionale dei Tumori, via Amadeo 42, 20113, Milano, Italy*

[3]*National Research Council of Italy Institute of Materials (CNR-IOM) c/o SISSA, via Bonomea 265, 34136 Trieste, Italy*

[4]*Department of Biological, Chemical and Pharmaceutical Sciences and Technologies, University of Palermo, Viale delle Scienze, 90128 Palermo, Italy*

** Correspondence: silvia.gobbi@unibo.it (S.G.); alessandra.bisi@unibo.it (A.B.)*

Contents

Figures S1 to S5: NMR spectra of compound **3b** p. S2-S6

Figures S6 to S9: NMR spectra of compounds **2a,b** and **4a,b** p. S7-S10

Figure S10: NMR spectra of intermediates **8a** and **8b** p.S11

Table S1 p. S12

NMR spectra of compound 3b

Figure S1. ^{1}H-NMR

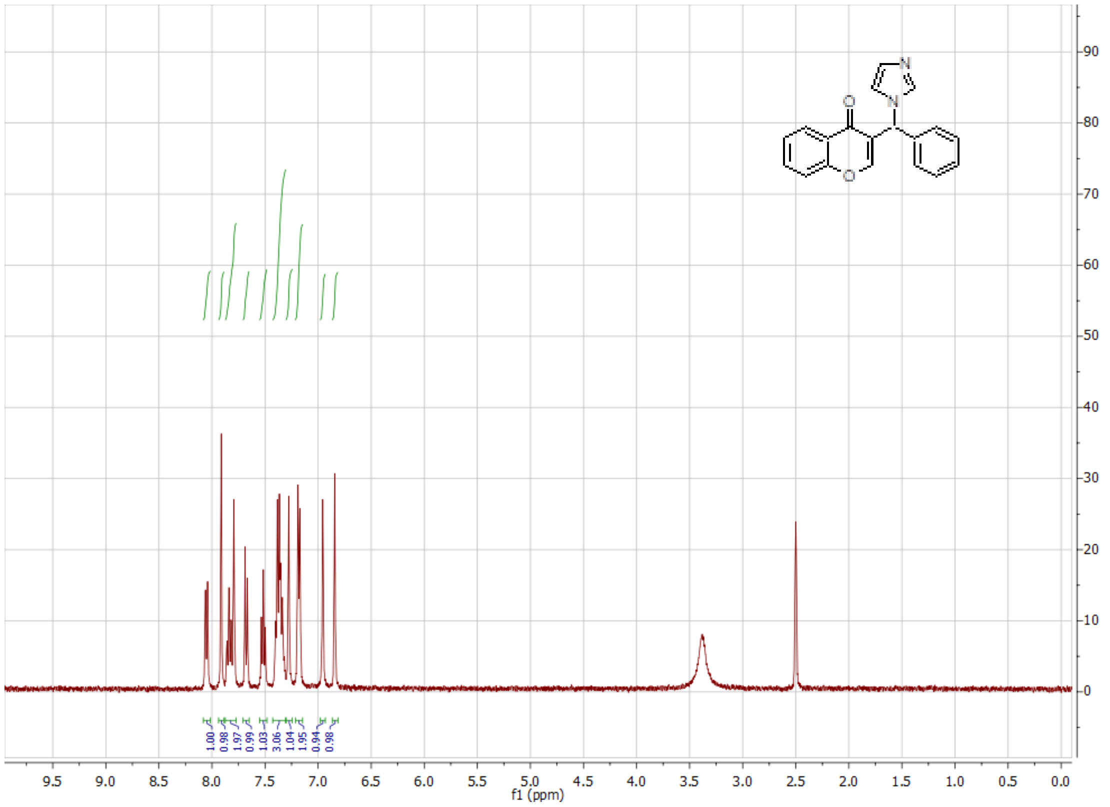

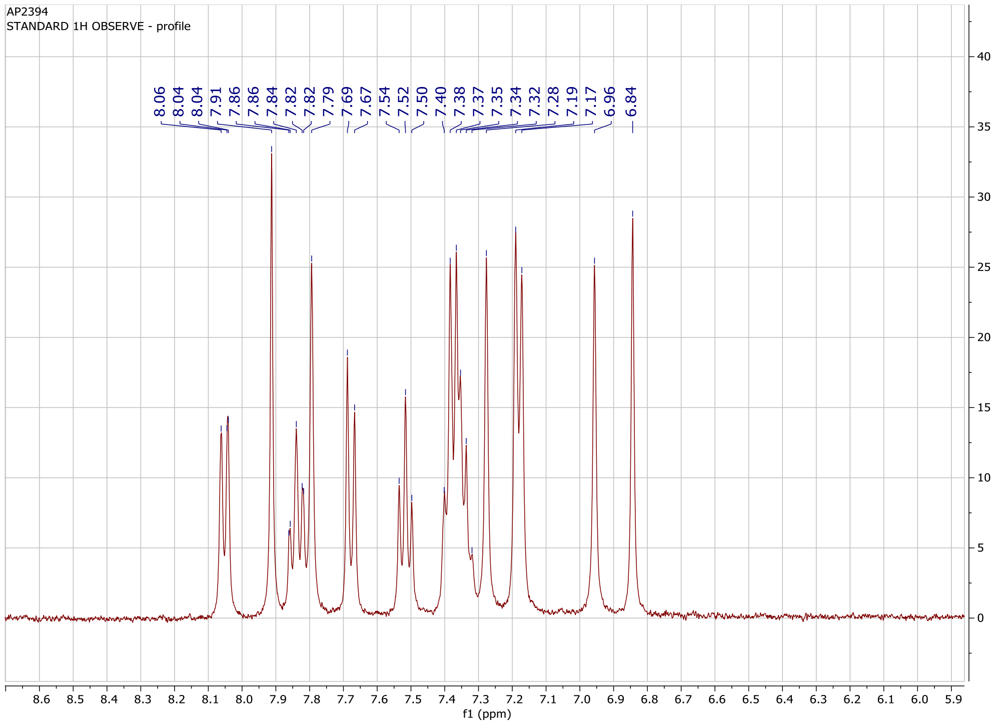

Expansion

Figure S2. ^{13}C-NMR

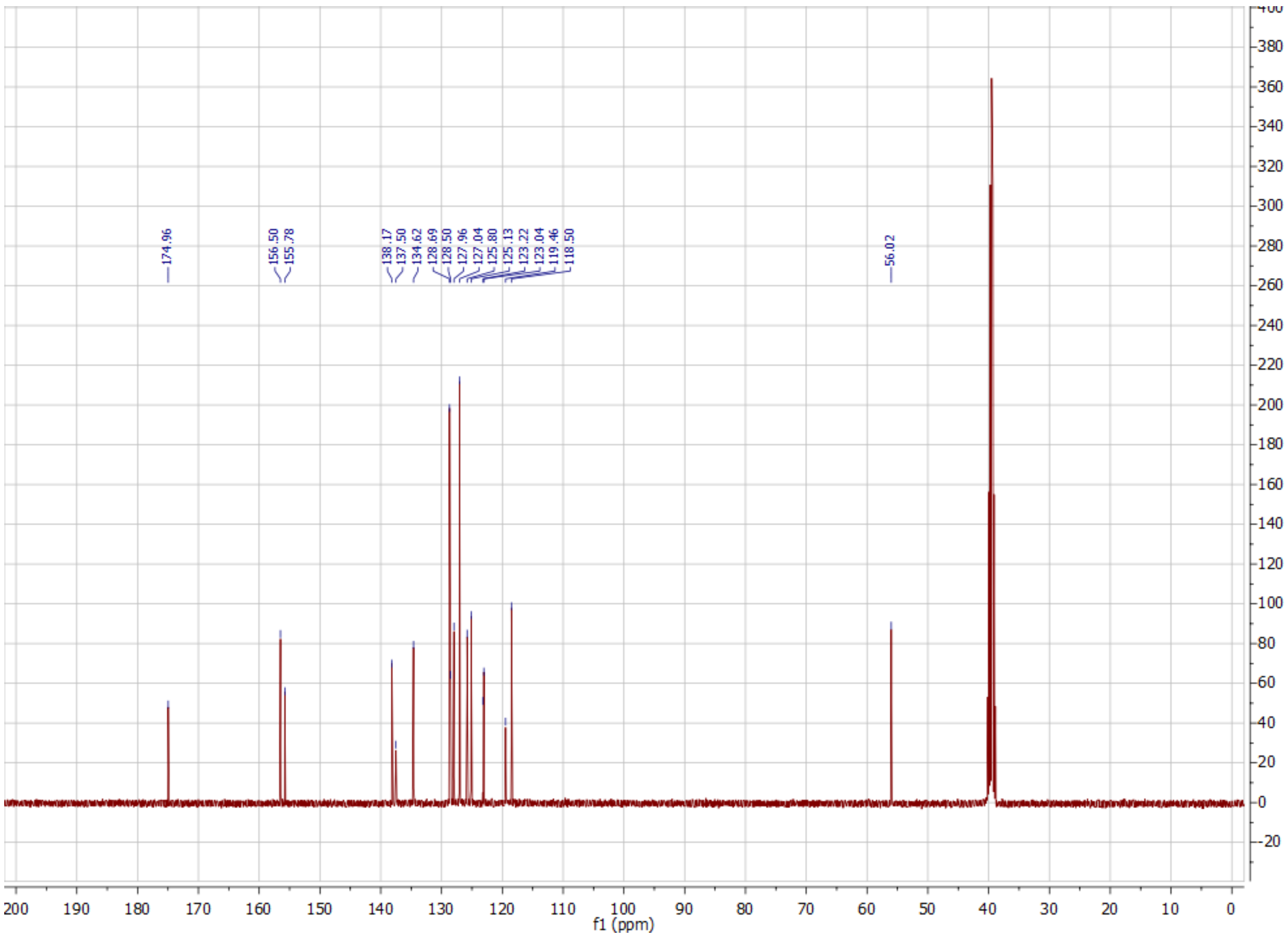

Figure S3. ^{1}H-^{1}H COSY

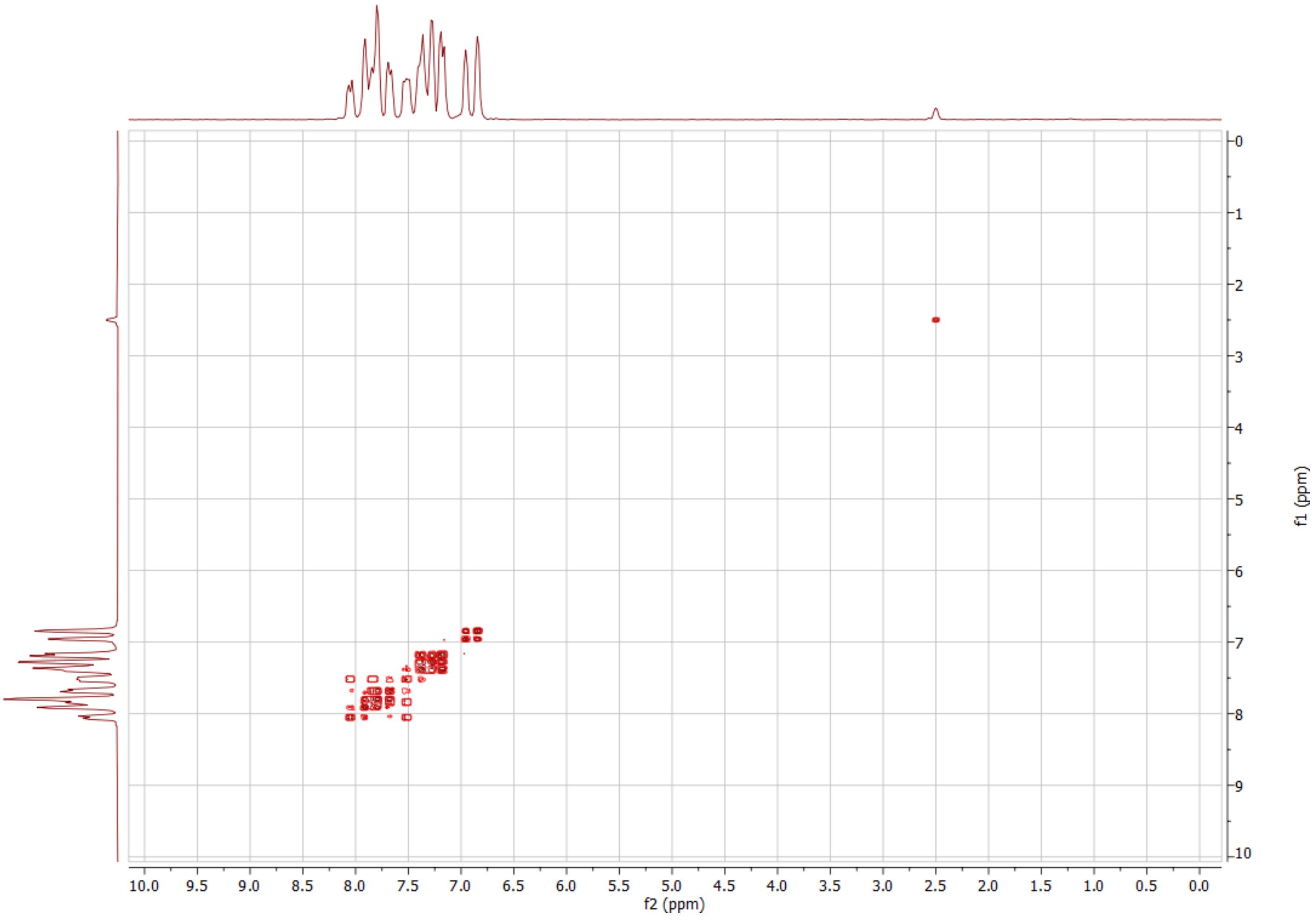

Figure S4. HMBC

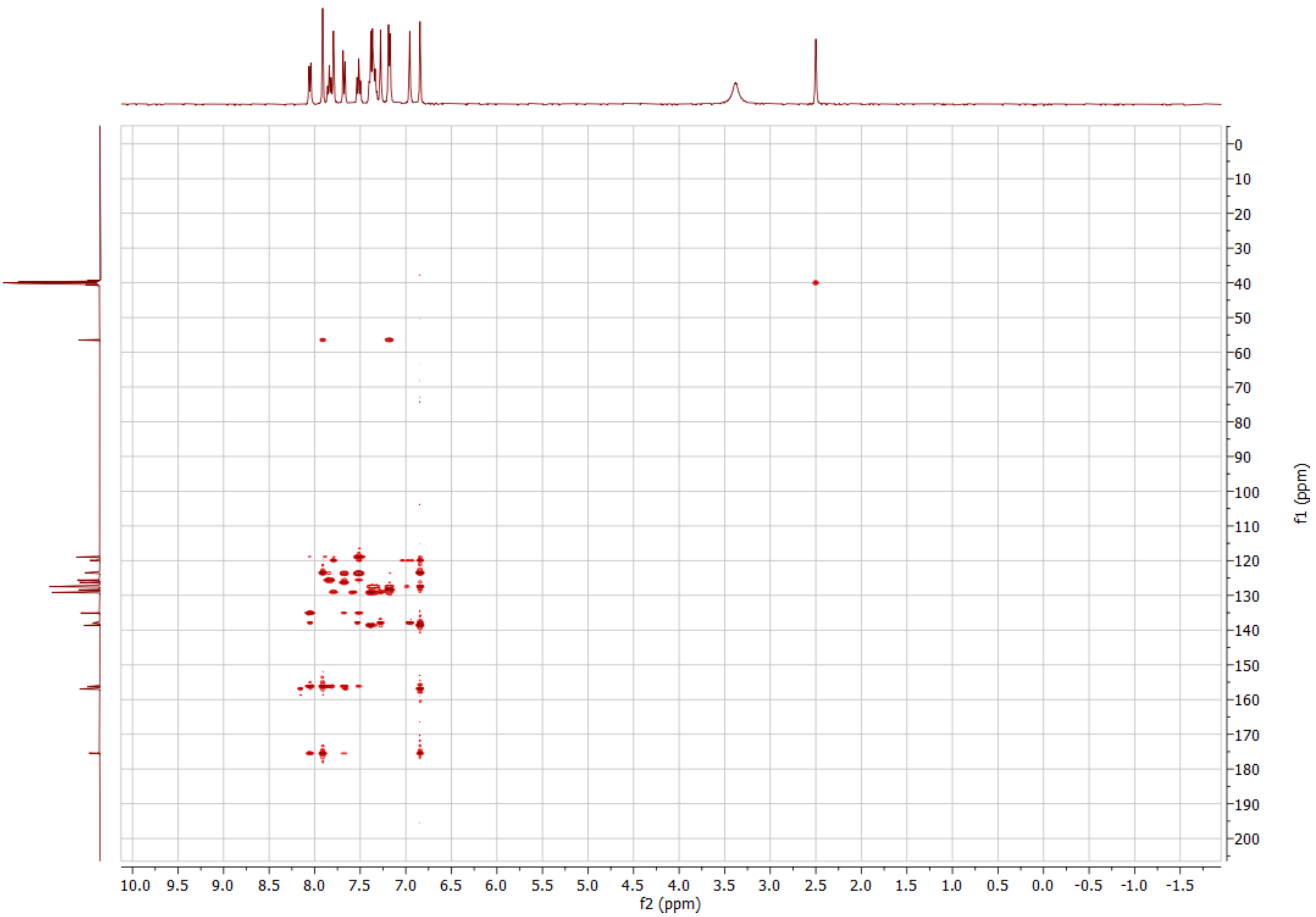

Figure S5. HSQC

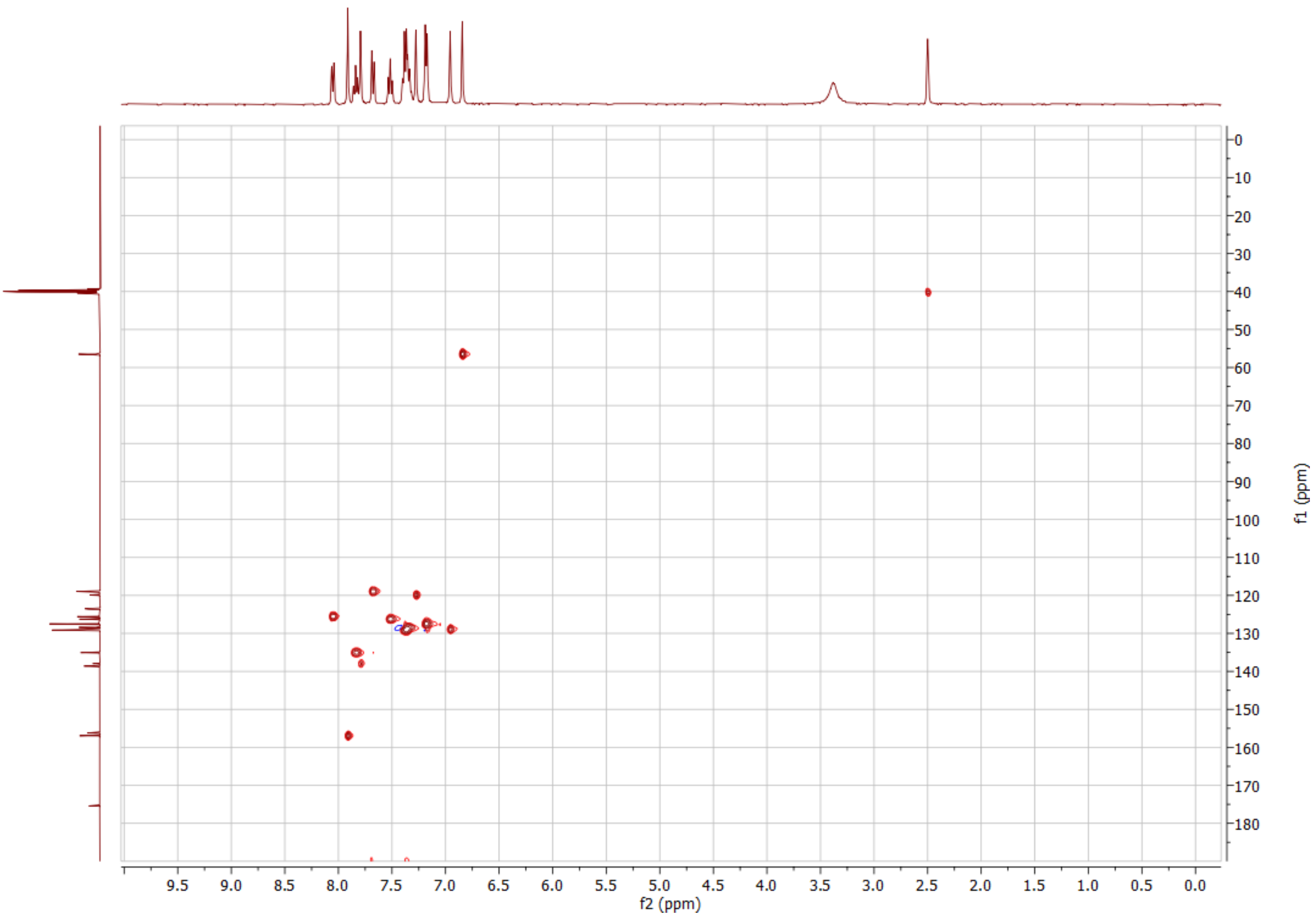

Figure S6. NMR spectra of 2a

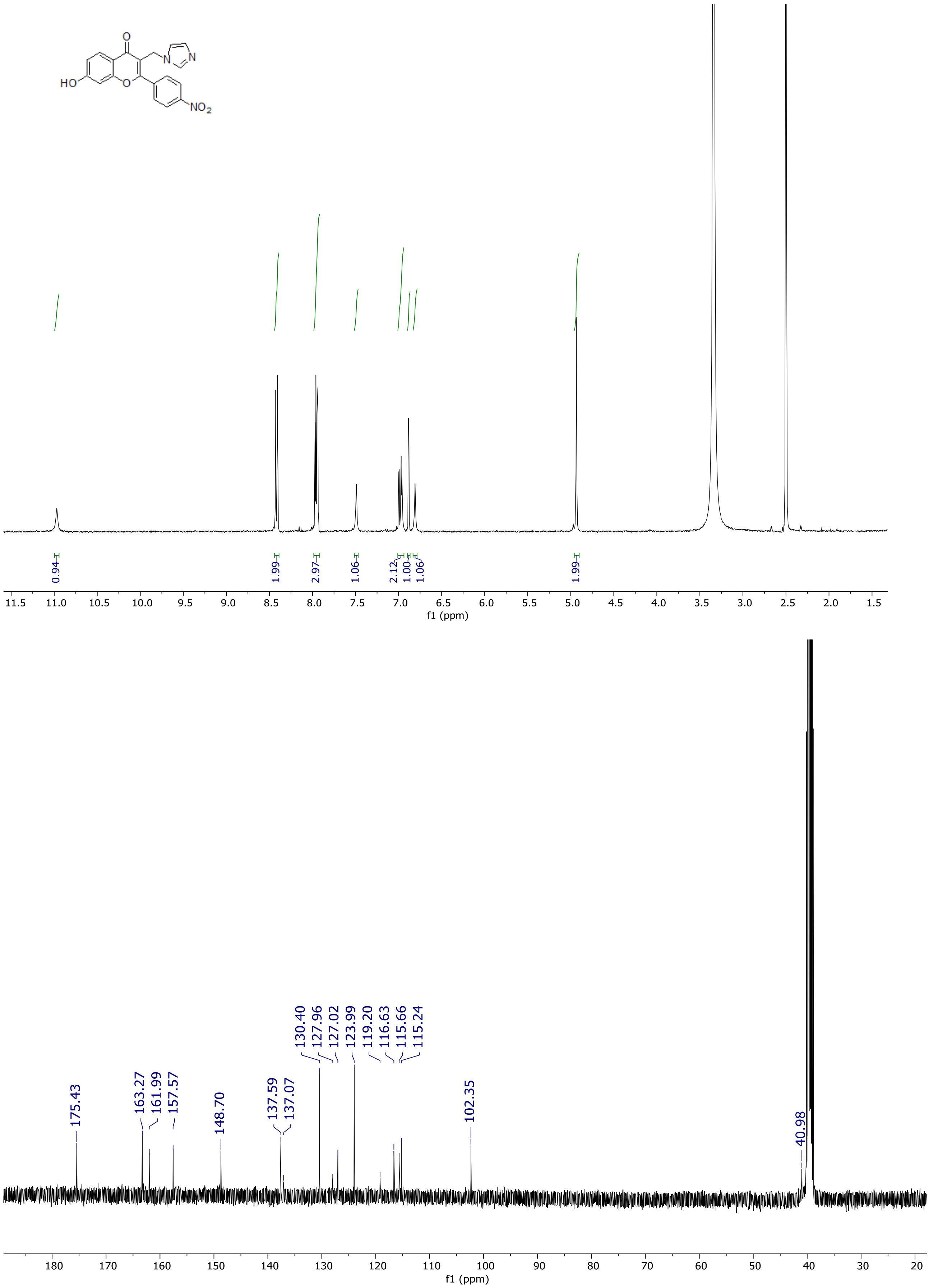

Figure S7. NMR spectra of 2b

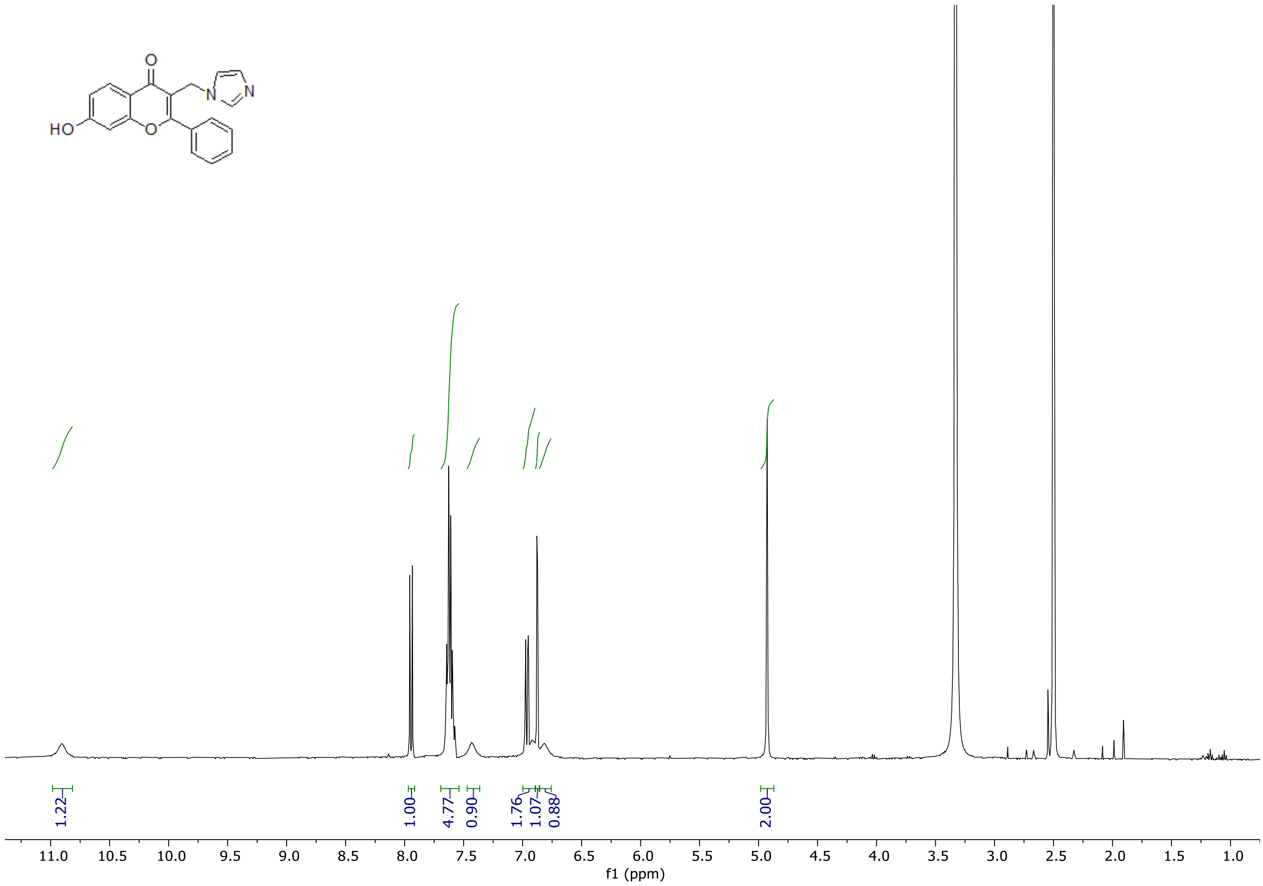

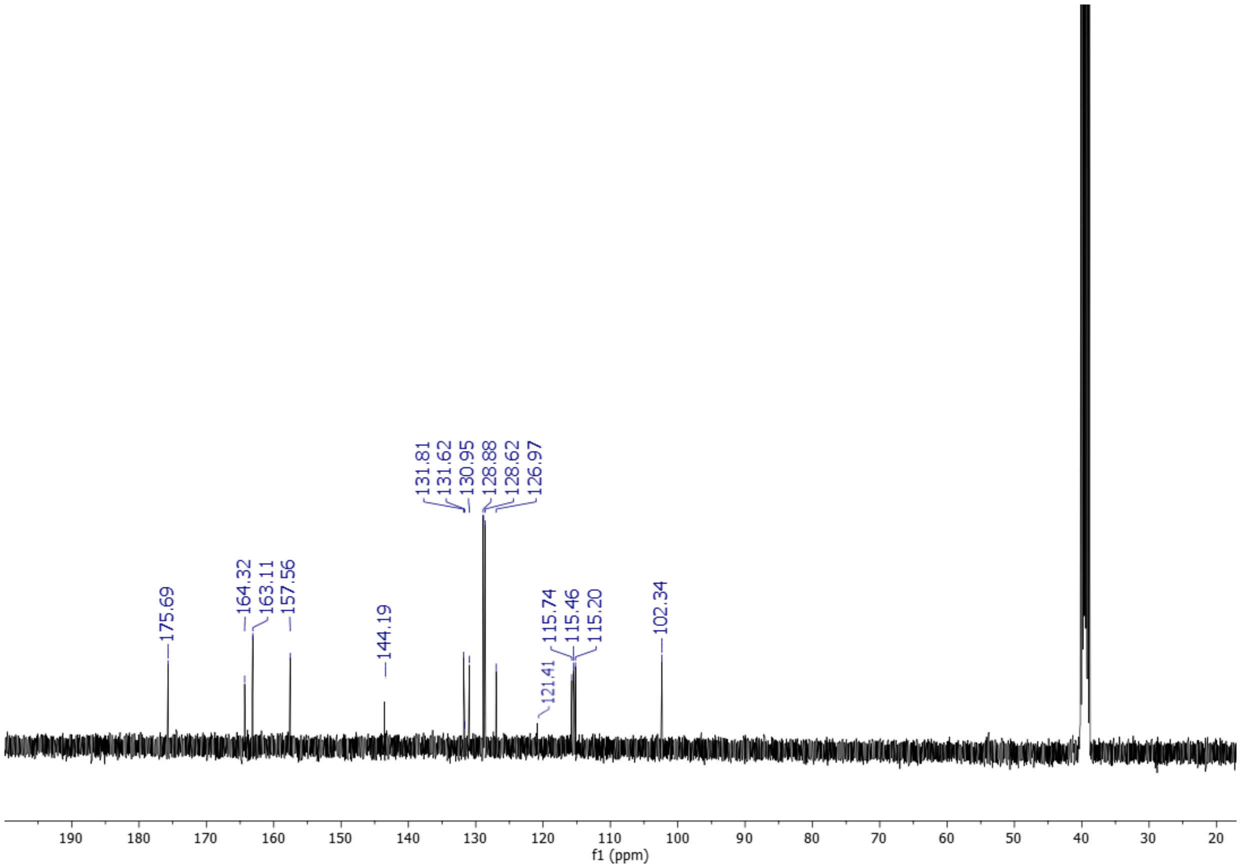

Figure S8. NMR spectra of 4a

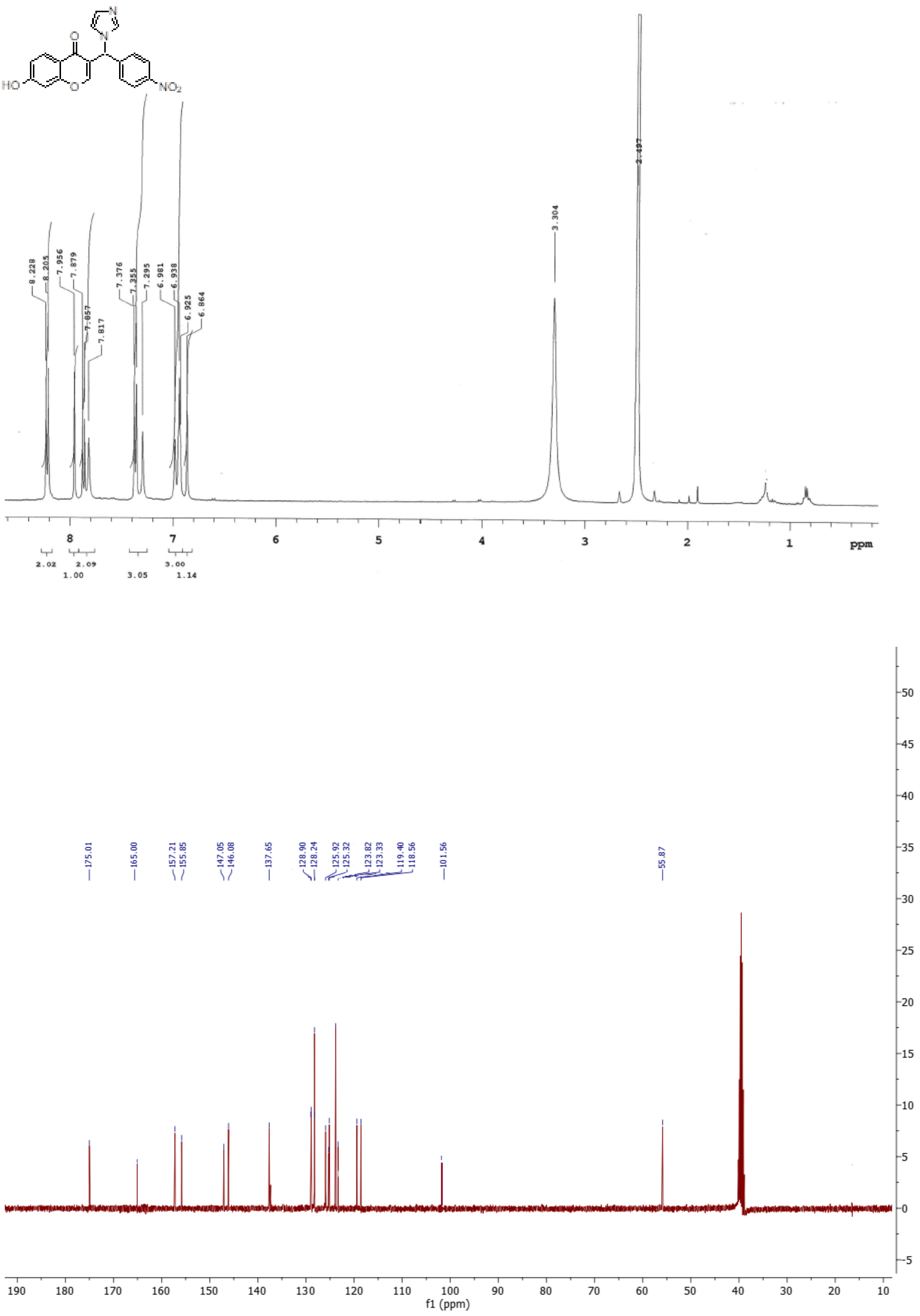

Figure S9. NMR spectra of 4b

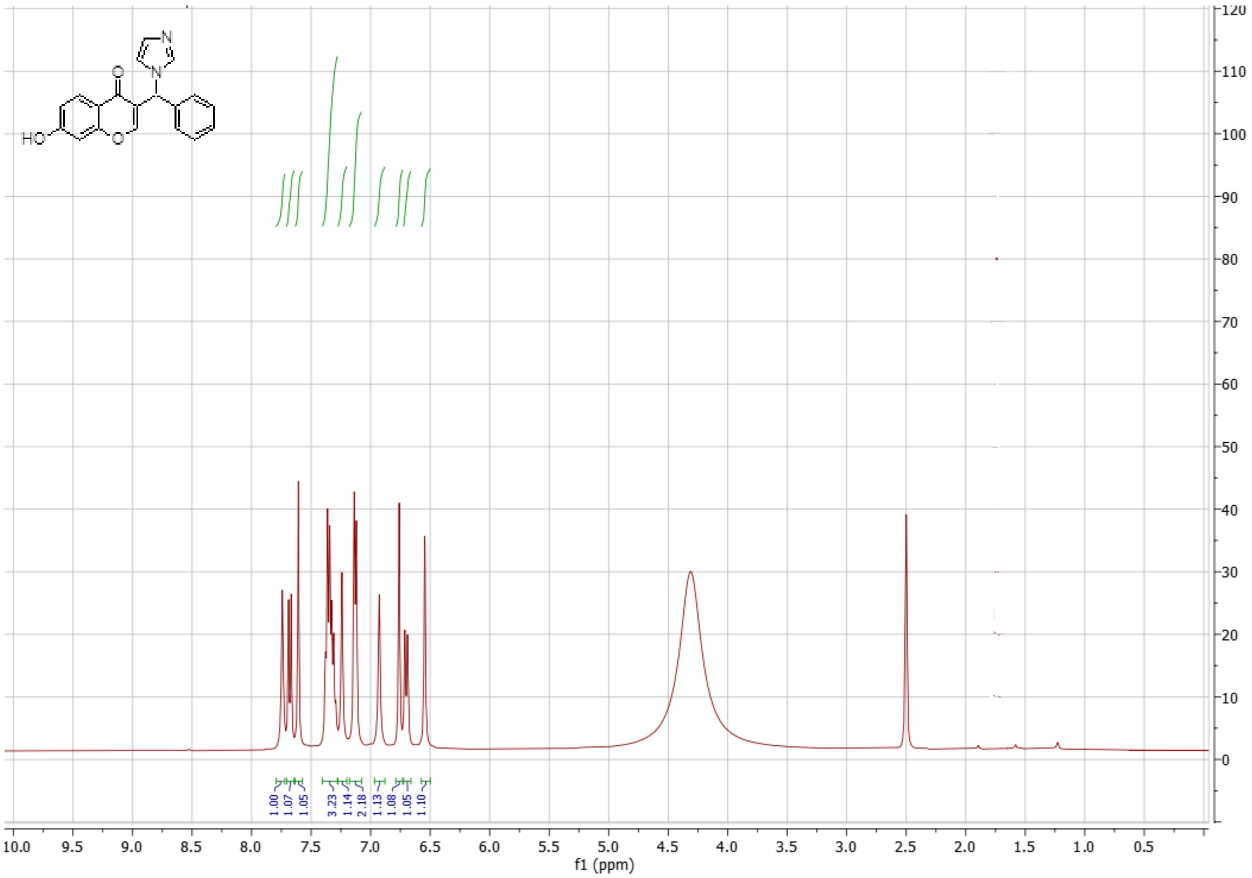

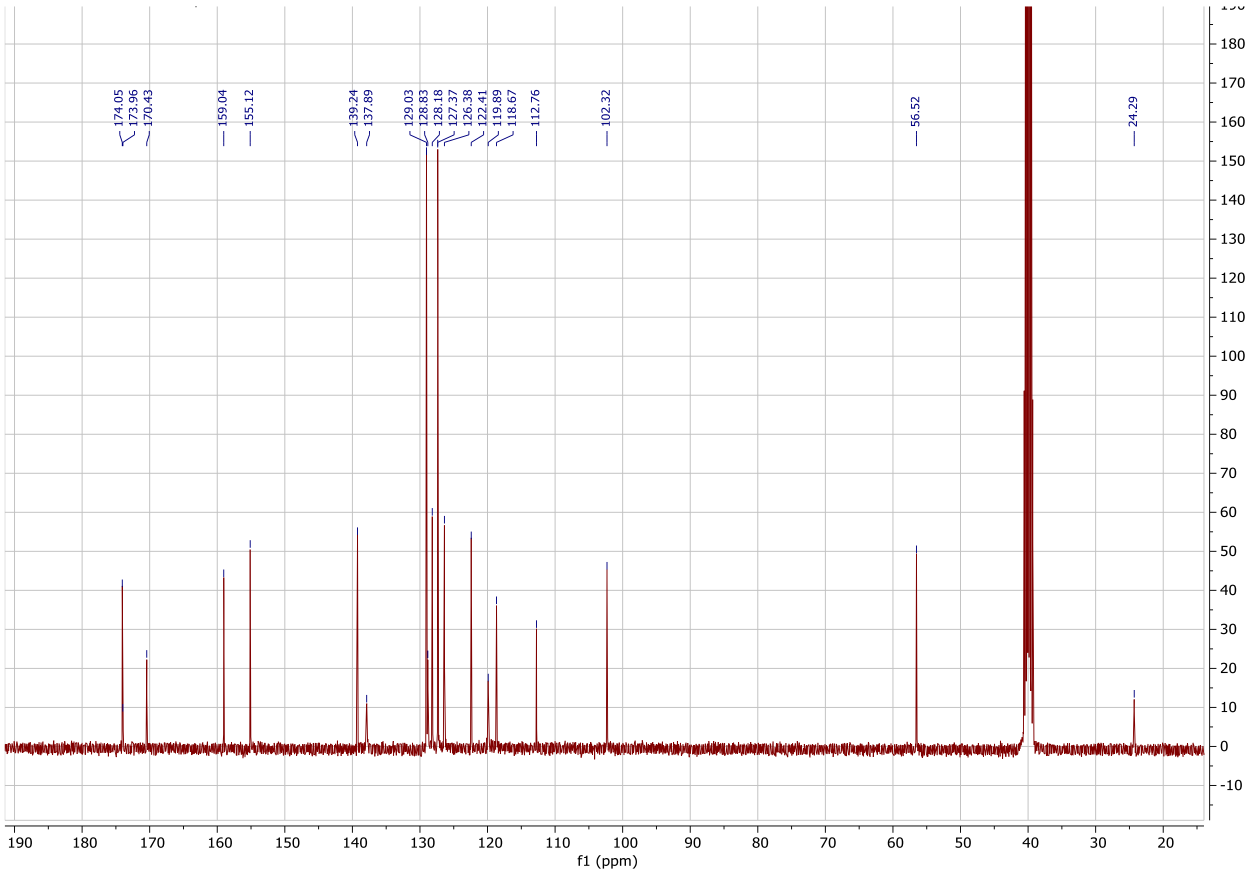

Figure S10. NMR spectra of intermediates 8a and 8b

8a

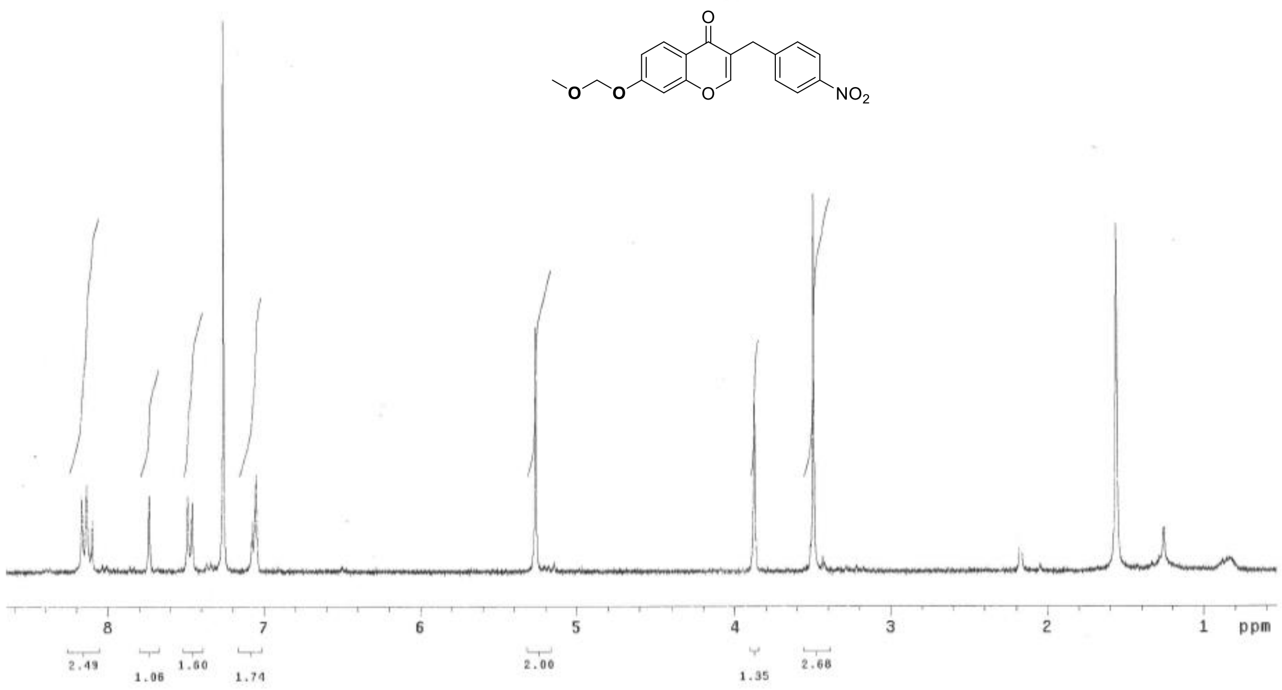

8b

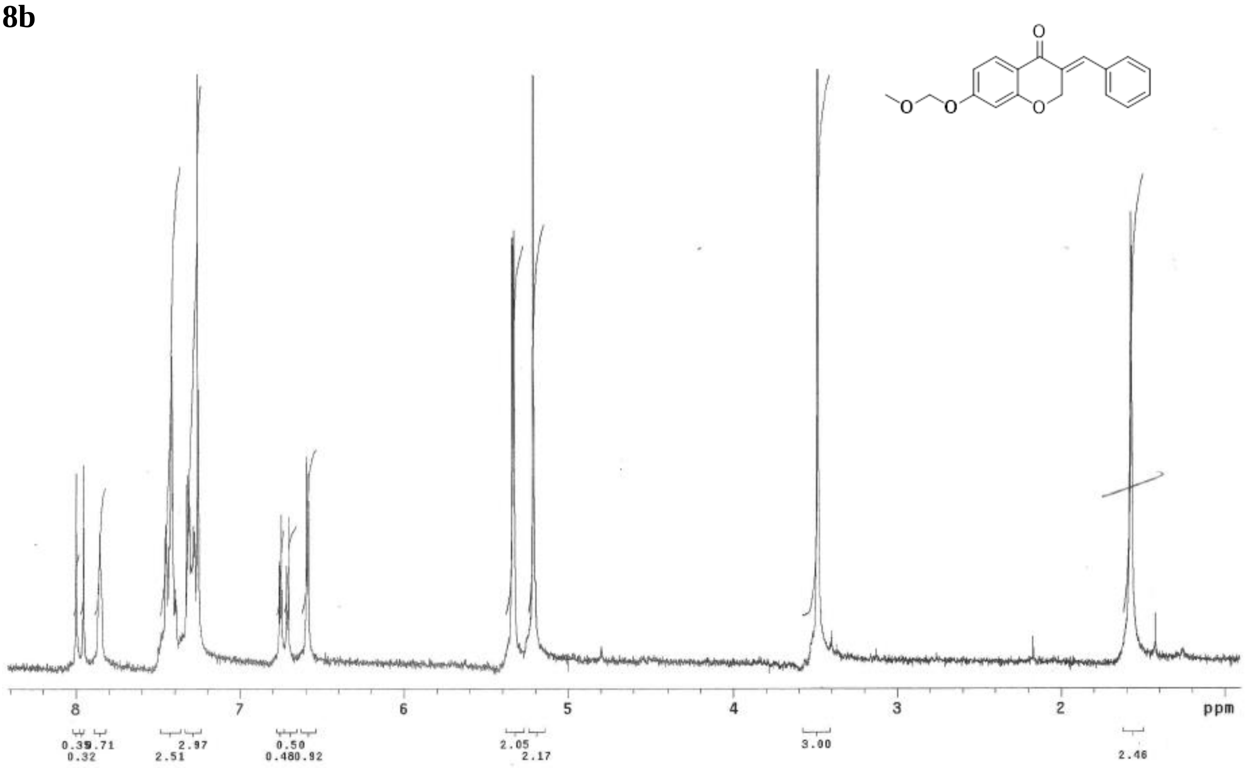

Table S1. Docking score (kcal/mol) to Estrogen Receptor α for the studied compounds. For compounds **3a,b** and **4a,b** we considered the R and S enantiomer in the docking simulations.

**Compound**	**Docking score**	**Docking score R**	**Docking score S**
**1a**	-9.76		
**2a**	-10.52		
**1b**	-9.85		
**2b**	-10.03		
**3a**		-9.53	-9.20
**3b**		-9.84	-8.68
**4a**		-10.49	-8.96
**4b**		-10.09	-10.38
